# AI‐Assisted Digital Single‐Molecule Activity Tracker for Decoupling Intrinsic Heterogeneity from Photo‐Oxidative Damage in High‐Photon‐Flux Enzymology

**DOI:** 10.1002/advs.76238

**Published:** 2026-06-18

**Authors:** Anran Zheng, Qi Yang, Jinze Li, Fuqiang Ma, Xuefeng Wang, Zhen Guo, Chuanyu Li, Dongshu Li, Jia Yao, Zhiqi Zhang, Wei Zhang, Lianqun Zhou

**Affiliations:** ^1^ CAS Key Lab of Bio‐Medical Diagnostics Suzhou Institute of Biomedical Engineering and Technology Chinese Academy of Sciences Suzhou China; ^2^ School of Biomedical Engineering (Suzhou) Division of Life Sciences and Medicine University of Science and Technology of China Hefei China

**Keywords:** deep learning, digital single‐molecule activity tracker, functional heterogeneity, long‐read sequencing, oxidative scarring, single‐molecule enzymology

## Abstract

Single‐molecule enzymes serve as molecular motors for long‐read sequencing, where laser tolerance under high photon flux is a critical limiting factor for ultra‐long reads. However, elucidating the mechanism of laser‐induced enzyme inactivation remains a technical bottleneck due to the lack of long‐term, high‐throughput single‐molecule evaluation methods to decouple intrinsic heterogeneity from photodamage. Here, a digital Single‐Molecule Activity Tracker (dSMAT) is presented, combining deep learning with high‐throughput digital microfluidics to enable the precision tracking of thousands of compartmentalized single‐molecule reactions for 15 h. This strategy reveals a distinct photoinactivation mechanism designated as oxidative scarring through comparative tracking of individual polymerases before and after laser irradiation. This process is driven by the stochastic accumulation of photochemical lesions on redox‐sensitive residues (specifically Methionine, Tryptophan, and Cysteine) within functionally accessible pathways, creating a kinetically disordered subpopulation. A synergistic reductive‐antioxidant buffer system is engineered to mitigate this effect and rescue kinetic homogeneity. Quantitative cross‐platform validation via single‐molecule real‐time sequencing confirms that dSMAT‐derived kinetic metrics—including catalytic rate, heterogeneity, and temporal stability—deterministically govern sequencing read limits. This work establishes a mechanistically sound biophysical framework for the rational design of photostable molecular motors, offering a generalizable strategy for enhancing high‐photon‐flux enzymology across genomic and biotechnological applications.

## Graphical abstract

Employing a digital single‐molecule activity tracker (dSMAT), this research demonstrates that high‐photon‐flux irradiation drives progressive oxidative scarring in polymerases. Unlike simple thermal denaturation, real‐time kinetic tracking dynamically visualizes enzymes degrading into multiple impaired subpopulations. Uncovering this concealed heterogeneity directly decodes stability bottlenecks, efficiently guiding mutant engineering and optimal buffer formulation for advanced biological recording systems.

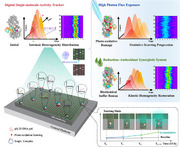

## 1 Introduction

Single‐molecule enzyme profiling yields valuable insights, including catalytic heterogeneity within the enzyme population and the proportion of functional peptides. These biopolymers exist in a variety of physical or chemical states, which correlate with distinct activity parameters [1, 2, 3]. This profiling is particularly critical for single‐molecule real‐time (SMRT) sequencing. Although this technology has driven the complete telomere‐to‐telomere (T2T) human genome and the pangenome draft, the technology inherently requires DNA polymerases to operate under intense laser irradiation that far exceeds their evolutionary tolerances [4, 5, 6]. Consequently, the laser tolerance of the enzyme has emerged as the definitive bottleneck constraining ultra‐long reads [7, 8]. Despite this urgency, the microscopic mechanism of laser‐induced failure remains a bottleneck: it has been technically challenging to decipher whether the motor halts due to cooperative thermal denaturation, structural fatigue, or a stochastic accumulation of chemical lesions [9, 10]. This knowledge gap severely impedes rational enzyme engineering, limiting the breakthrough of read length in existing optical sequencing systems [11, 12].

However, deciphering this failure mechanism is impeded by a lack of single‐molecule enzyme functional activity evaluation methods capable of long‐term, high‐throughput analysis, preventing the decoupling of intrinsic polymerase heterogeneity from laser‐induced photodamage [13]. Traditional bulk biochemical techniques (e.g., circular dichroism, nuclear magnetic resonance, mass spectrometry inherently obscure single‐molecule heterogeneity in catalytic activity [14, 15, 16, 17], synthesis stability, and conformational dynamics [18]. Current single‐molecule techniques face intrinsic limitations: single molecule fluorescence resonance energy transfer (smFRET) provides high‐resolution conformational data but is constrained by short observation windows [19]; atomic force microscopy (AFM) characterizes mechanics but offers limited temporal resolution for monitoring real‐time kinetics or fluorescence [20, 21]; optical tweezers offer high precision but display low throughput and high system complexity [22, 23, 24]; molecular circuits typically lack the capacity for long‐term multidimensional dynamic analysis [25, 26]. Similarly, nanopore sensing cannot achieve laser‐related multiparametric fluorescence analysis [27, 28]. Although commercial zero‐mode waveguide (ZMW) platforms are effective, they are closed and cost‐prohibitive systems unsuited for mechanistic dissection, limiting their routine use in polymerase evolution and buffer optimization [29]. Crucially, most existing studies focus on fluorophore bleaching rather than enzyme inactivation [23]. Furthermore, conventional bulk assays are physically unequipped to decouple binary denaturation from heterogeneous kinetic deceleration. Thus, there is an urgent need for open, high‐throughput, and cost‐effective platforms to characterize the multidimensional kinetic profiles of single polymerases under physiological photooxidative conditions.

In this work, the digital Single‐Molecule Activity Tracker (dSMAT) was developed as a photodamage evaluation strategy combining the robustness of deep learning with the high‐throughput capabilities of digital microfluidic platforms, which have proven highly effective in resolving complex biological heterogeneity [30]. By compartmentalizing rolling circle amplification (RCA) reactions in picoliter wells, we utilize a custom AI pipeline to correct mechanical drift and track thousands of single‐enzyme trajectories for over 15 h. This approach enables the extraction of a multidimensional kinetic signature: the catalytic synthesis rate (*µ*, serving as the primary proxy for polymerization velocity), inter‐molecular heterogeneity (*σ^2^*), temporal synthesis variation (*θ*, capturing processive stability), and apparent kinetic efficiency (*K_app_*). Crucially, by tracking the long‐term kinetic changes of individual polymerases before and after laser irradiation, a distinct failure mode termed oxidative scarring was discovered. Our data suggest that photo‐activated reactive oxygen species (ROS) stochastically target sensitive residues within functional protein cavities—a mechanism we propose as the primary driver of kinetic spectral broadening, rather than bulk thermal denaturation. This impairment is effectively mitigated via a synergistic reduction–antioxidant buffer system. The strong concordance with single‐molecule real‐time (SMRT) sequencing results (equivalent error < 1.0%) demonstrates the high accuracy and predictive value of dSMAT. Collectively, our study establishes a mechanistically sound framework for designing sequencing enzymes with enhanced photostability and extended read lengths.

## 2 Results

### 2.1 Design of Dynamic Multi‐Activity Characteristics Evaluation Strategy

**FIGURE 1:**
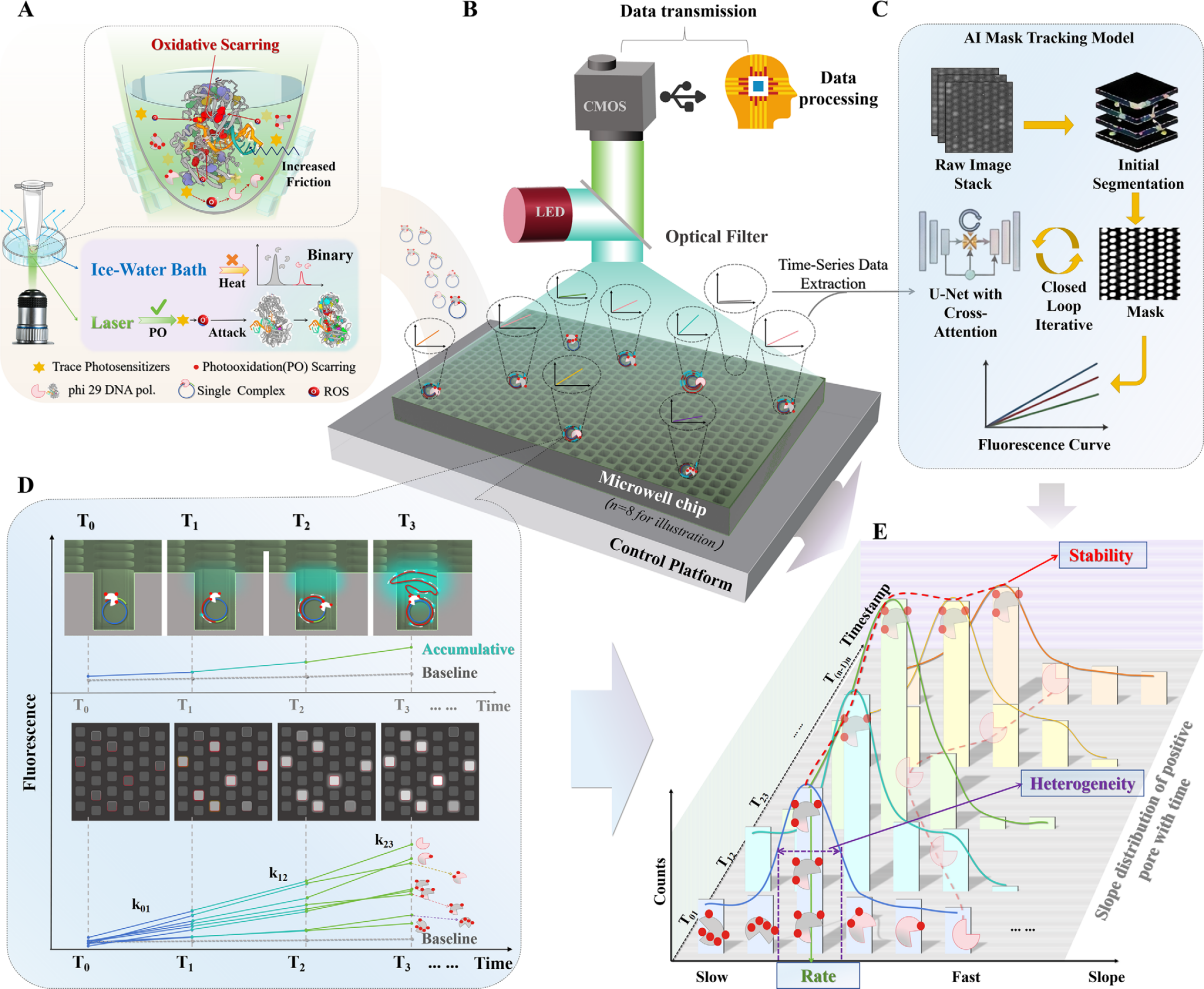
Design of the AI‐assisted dSMAT strategy. (A) Simulation of photo‐oxidative stress mechanisms. Enzyme samples are subjected to high‐intensity laser irradiation in an ice‐water bath to investigate the oxidative scarring mechanism. Trace photosensitizers mediate ROS generation, creating a heterogeneous library of functionally impaired complexes. (B) Hardware architecture of the dSMAT platform. The system integrates a high‐density microwell chip for single‐molecule partitioning with a custom LED‐CMOS optical setup. Data transmission couples real‐time imaging with an AI‐assisted processing unit. (C) Intelligent fluorescence extraction module. The original image sequence is segmented via a U‐Net architecture with an attention mechanism to track and generate accurate microwell masks, achieving precise extraction of fluorescence values within the wells. (D) Real‐time signal quantification. Inside the microwells (*n* = 8 for illustration), single polymerase molecules drive RCA. As the reaction proceeds from *T_0_* to *T_3_*, fluorescence accumulation is monitored. The individual relative catalytic rate is derived as the calibrated fluorescence slope (*k*) from intensity trajectories (*k_01_*, *k_12_*). (E) Construction of the dynamic multi‐activity landscape. A 3D statistical distribution of catalytic rates is generated across the time course (*T_01_* to *T_(n−1)n_*). This visualization simultaneously resolves three core functional characteristics: catalytic synthesis rate (*µ*, distribution peak center), inter‐molecular kinetic heterogeneity (*σ^2^*, distribution variance), and temporal synthesis variation (*θ*, trajectory fluctuation).

**FIGURE 2:**
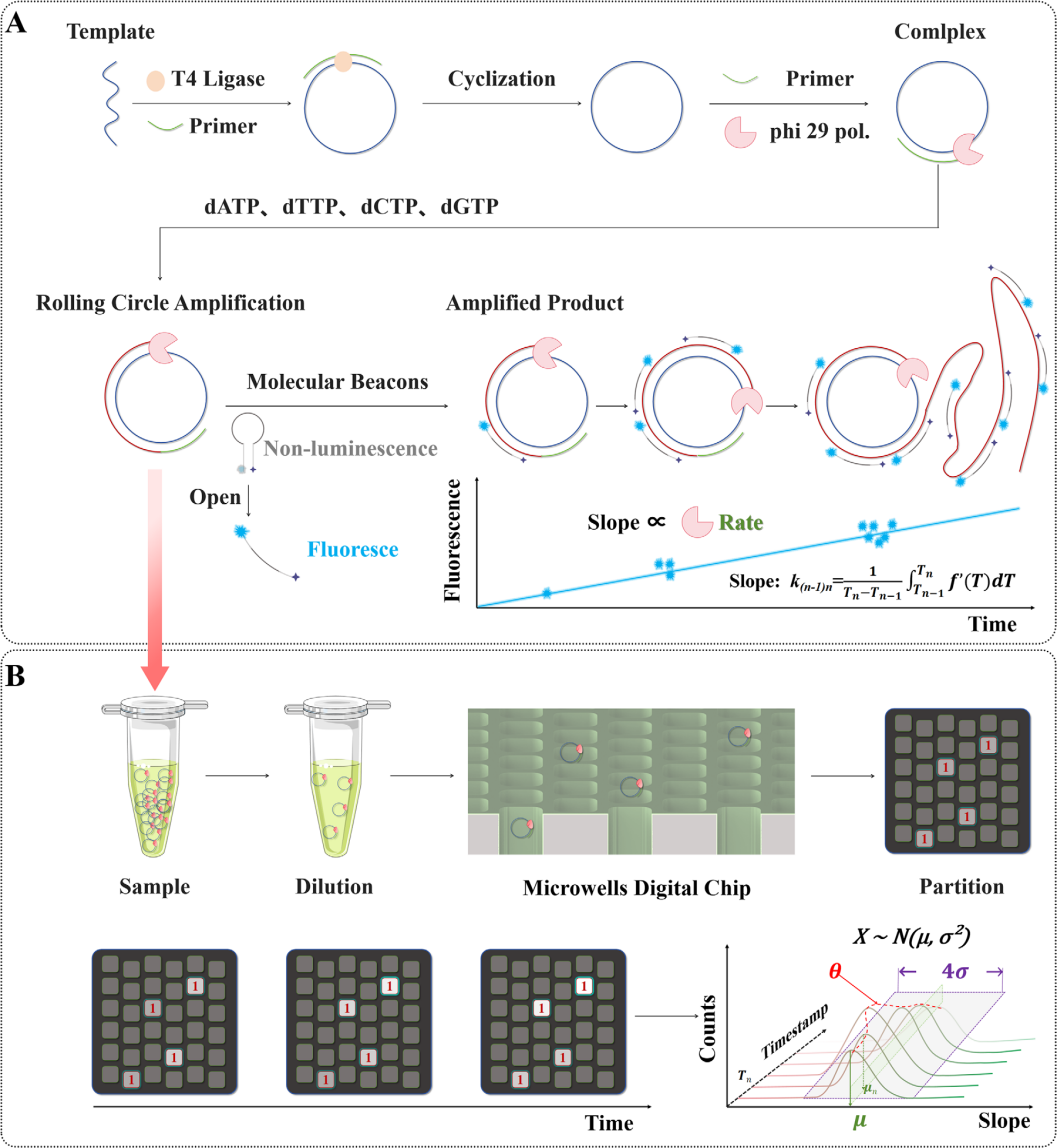
The principle of the dSMAT method. (A) Schematic of the RCA reaction mediated by phi29 DNA polymerase. The molecular beacon, which is initially non‐fluorescent (in the closed state), hybridizes with the nascent amplification product to emit fluorescence. The slope of the fluorescence intensity over time is proportional to the enzymatic catalytic rate. (B) Single‐molecule compartmentalization. Enzyme‐template complexes are subjected to limiting dilution and stochastically partitioned into the high‐density microwell chip. Single enzymes catalyze amplification within individual wells (positive pores), generating fluorescence. As the reaction progresses, the statistical distribution of fluorescence slopes enables the multidimensional evaluation of catalytic rate, uniformity, and temporal synthesis variation at the single‐molecule level.

The design of the dSMAT single‐molecule enzyme characterization strategy, based on a digital microfluidic chip, is illustrated in Figure 1. The assay relies on monitoring fluorescence accumulation within a molecular beacon‐based RCA system [31, 32, 33, 34]. To ensure predominantly single‐molecule compartmentalization, enzyme‐template complexes were diluted to satisfy Poisson statistics (*λ* = 0.15) prior to loading into the microwell array (Figure 2B; Figure S1A) [35].

By applying an AI‐assisted analysis pipeline to these kinetic trajectories, we simultaneously quantified the catalytic rate (*µ*), heterogeneity (*σ^2^*), and temporal variation (*θ*). The underlying principles and calculation equations are detailed in the Methods section. Notably, a lower *θ* indicates lower temporal synthesis variation, and a lower *σ^2^* value indicates reduced catalytic dispersion (or narrowing of intermolecular diversity) across the enzyme population, a feature crucial for applications requiring high processive consistency, such as long‐read SMRT sequencing [36, 37].

Furthermore, to deconvolute the stochastic fluctuations inherent to single‐molecule catalysis, we establish two supplementary metrics: the apparent kinetic efficiency index (*K_app_*) and the cumulative turnover number (*P*). *K_app_* acts as a unified proxy for the enzyme's instantaneous productive state relative to its theoretical maximum (*V_max_*). Time‐resolved analysis of *K_app_* trajectories exposed profound differences in conformational dynamics otherwise masked by population averaging [38, 39]. *P* integrates the instantaneous rate over the total reaction duration to project the total nucleotide yield. By integrating the instantaneous rate, this metric (*P*) effectively bridges the gap between in vitro kinetics and genomic output. It indicates that enzymes with lower kinetic heterogeneity (*σ^2^*) can sustain higher cumulative turnovers, thereby generating longer continuous reads in sequencing applications by minimizing early termination events.

### 2.2 Image Processing and Data Analysis by AI‐Assisted Models

In long‐term time‐lapse analysis, the weak cumulative nature of fluorescence signals determines that detection stability is directly related to the confidence and reliability of analytical results. Under high‐magnification imaging conditions, vibrations generated by pressure pump operation and minor environmental disturbances are significantly amplified into pixel‐level displacements. The conventional fixed‐template sampling method suffers from two critical limitations: on one hand, it fails to accurately track target regions due to well displacement, leading to sampling bias and signal truncation; on the other hand, to avoid interference from stray signals outside the wells, statistical filtering must be applied to raw signals, which discards valid intraluminal signals and further reduces the signal‐to‐noise ratio.

To address these issues, this study developed a dSMAT‐AI algorithm. Drawing on spatiotemporal denoising and multi‐dimensional tracking methodologies [40, 41], this algorithm is specifically designed to tolerate physical instability in experimental systems, with a core strategy of iterative recursion and precise segmentation, based on the key assumption that inter‐frame microchamber displacement is always constrained within the microchamber radius. Specifically, the centroid coordinates of the microchamber in frame *t* serve as spatial prompts (point vectors) to provide prior information for localizing the same chamber in frame *t+1*, thereby achieving cross‐frame chamber tracking.

**FIGURE 3:**
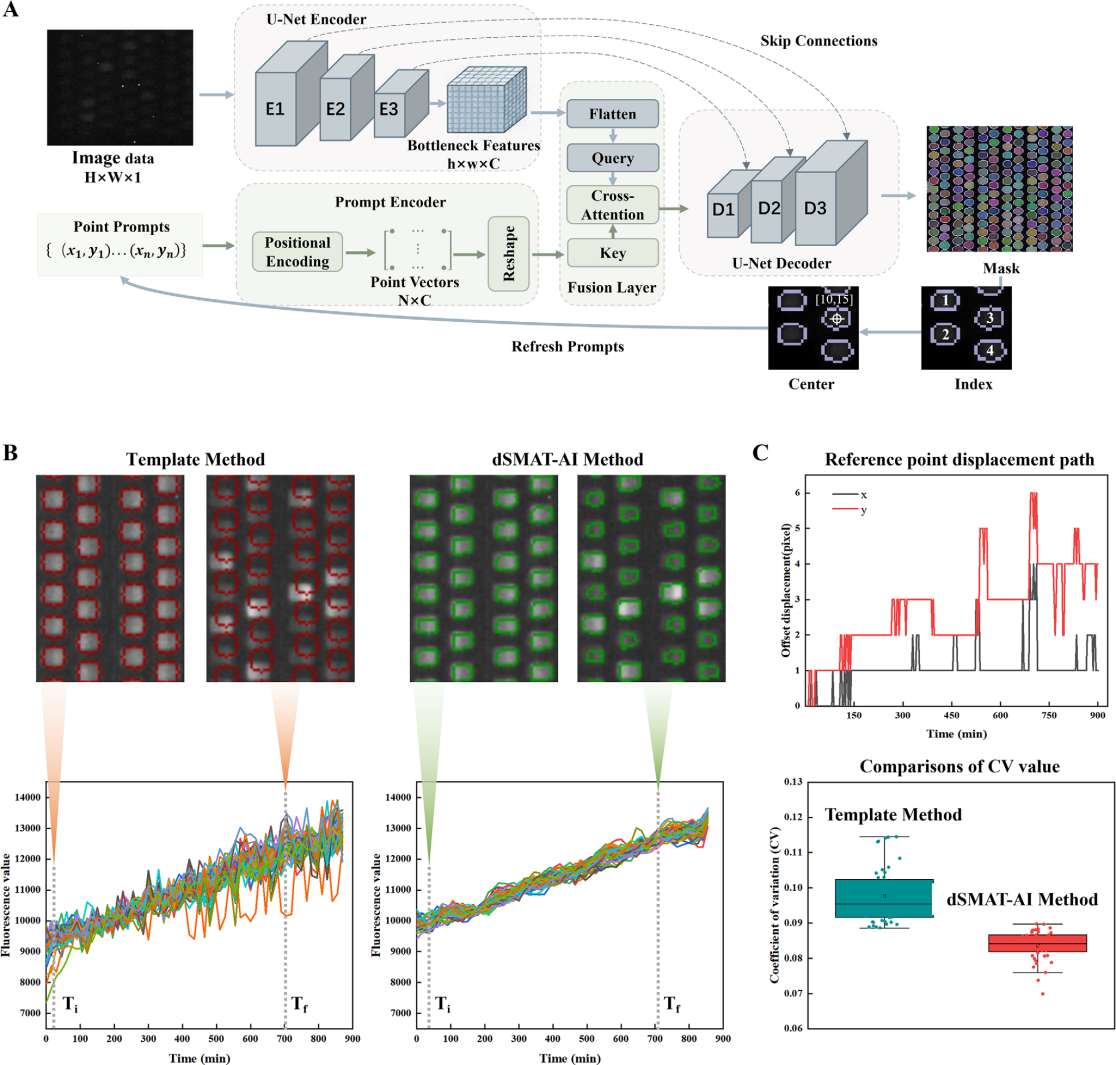
dSMAT‐AI—deep learning‐based ROI extraction method. (A) Point prompt‐based U‐Net algorithm for microwell tracking. The image of the first frame and the initial microwell coordinates are simultaneously input into the model to generate position‐specific masks. The centroids of these generated masks are calculated and serve as point prompts for the subsequent frame. This process is iterated to obtain microwell masks for all experimental cycles, followed by fluorescence intensity extraction. (B) Top: Comparison of the segmentation contours generated by the template method versus the AI Mask Tracking model at *T_1_* and *T_f_*; Bottom: Comparison of fluorescence signals extracted using the two methods. (C) Top: Temporal trajectory of the absolute position (x and y coordinates) of a reference microwell during the entire experiment; Bottom: Box plot comparison of the coefficient of variation (CV) of the fluorescence signal between the two methods.

The algorithm architecture employs U‐Net as the backbone network [42] (Figure 3A) and integrates a cross‐attention mechanism [43]. Spatial coordinates are embedded into visual features, and the fused representation of “spatial prompts + visual features” guides the network to precisely target microwells, enabling robust tracking even when microwells drift away from their initial positions. By iteratively computing the mask centroid and dynamically updating positional prompts for subsequent frames, the algorithm establishes a closed‐loop drift correction system: on one hand, the region of interest (ROI) dynamically adapts to physical shifts of the chip; on the other hand, the dynamic mask fully captures intraluminal fluorescence signals. Experimental results demonstrate that, compared with the traditional template method, the proposed algorithm significantly improves the integrity of signal capture and effectively enhances the signal‐to‐noise ratio. In a comparison of 200 sampling wells within the same batch, both the coefficient of variation (CV) and standard deviation were greatly reduced (Figure 3C).

### 2.3 Specificity and Feasibility Validation of Multi‐Activity Assay of phi29 DNA Polymerase Based on dSMAT Strategy

**FIGURE 4:**
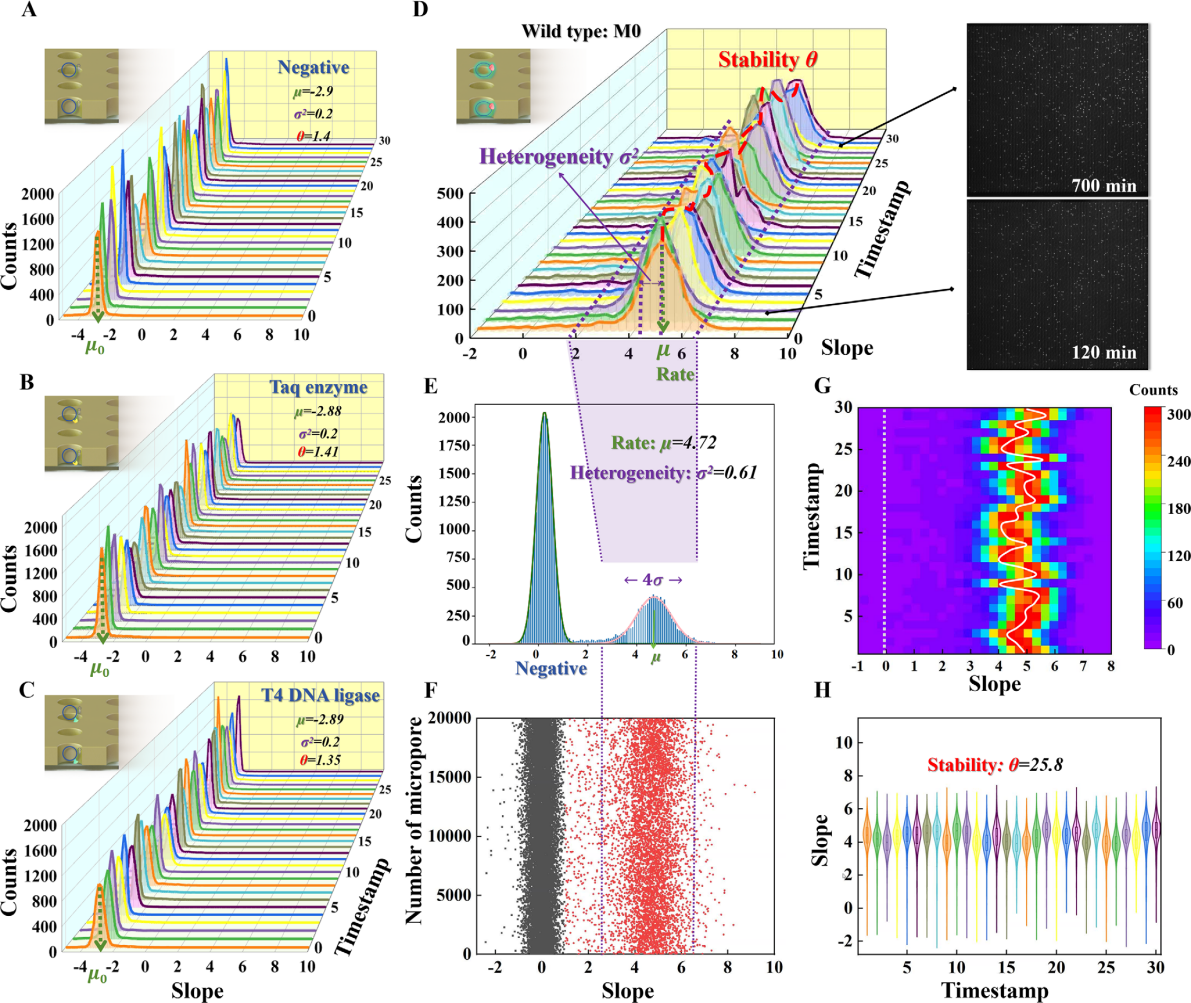
Specificity and feasibility validation of the dSMAT method. Experiments were performed using wild‐type phi29 DNA polymerase unless otherwise specified. (A–C) Real‐time fluorescence analysis of negative controls and non‐strand‐displacing enzymes. (A) Negative control (no enzyme) showing baseline activity. (B) Taq DNA polymerase (non‐strand‐displacing), exhibiting minimal background synthesis. (C) T4 DNA ligase control demonstrating negligible polymerization activity. All control conditions show significantly reduced signal parameters. Note that the *µ* values annotated in panels A–C strictly denote the statistical expectation center of the instrumental background optical drift (equivalent to the uncalibrated zero‐state *k_raw_* baseline), acting as the quantitative noise floor for authentic activity subtraction. (D–H) Temporal evolution of the slope distribution in positive microwells for wild‐type polymerase M0 based on dSMAT analysis. (E) Slope distribution statistics for the M0 enzyme over 900 min. (F) Clustered scatter plot of the slope distribution for the M0 enzyme over 900 min. (G) Spatiotemporal heatmap showing the variation in M0 enzyme slopes over time (30‐min intervals). (H) Violin plot of the M0 enzyme slope distribution over time (*n* > 1000 active wells per condition).

To benchmark the specificity of the dSMAT platform for capturing strand‐displacement synthesis, we performed rigorous negative control experiments involving non‐strand‐displacing polymerases (Taq Pol), T4 DNA ligase, and enzyme‐free conditions. As anticipated, all three control groups exhibited negligible catalytic activity, characterized solely by basal instrument noise and non‐enzymatic fluorescence probe drift. Although the global statistical algorithm fits a unified probability distribution to these blank microwells (yielding a mathematical mean mathematically annotated as *µ* = −2.90 in Figure 4A–C), this value functionally represents the pure background raw decay trajectory (*k_raw_*). This zero‐activity baseline extraction effectively establishes a clean mathematical noise floor, ensuring that signals collected in experimental groups are amplification events rather than non‐specific probe hybridization or aggregation artifacts.

Following specificity validation, the platform's quantitative feasibility was assessed using wild‐type phi29 DNA polymerase (M0). In contrast to the baseline of controls, the M0 reaction yielded a robust population of active microwells with positive fluorescence slopes. Crucially, rather than yielding a single population average, dSMAT resolved the intrinsic functional heterogeneity of the enzyme population. The single‐molecule rate distribution reveals a broad Gaussian profile (heterogeneity *σ^2^* = 0.61), quantitatively demonstrating the inherent kinetic asynchrony among individual phi29 molecules (Figure 4D–F).

Furthermore, the high temporal resolution allowed us to quantify temporal synthesis variation. By tracking the rate trajectory of individual enzymes over 900 min, we derived an average coefficient of variation (*θ*) of 25.8% (Figure 4G–H). This metric mathematically quantifies the continuity of single‐molecule synthesis, effectively capturing kinetic deviations driven by stochastic pausing and early termination. The ability to simultaneously extract the synthesis rate (*µ* = 4.72), intermolecular heterogeneity (*σ^2^*), and temporal variation (*θ*) from the same dataset validates dSMAT as a multi‐dimensional profiling tool capable of capturing complex enzymatic behaviors obscured in ensemble averaging.

### 2.4 High‐Throughput Profiling of Mutant and Commercial Polymerase Variants

**FIGURE 5:**
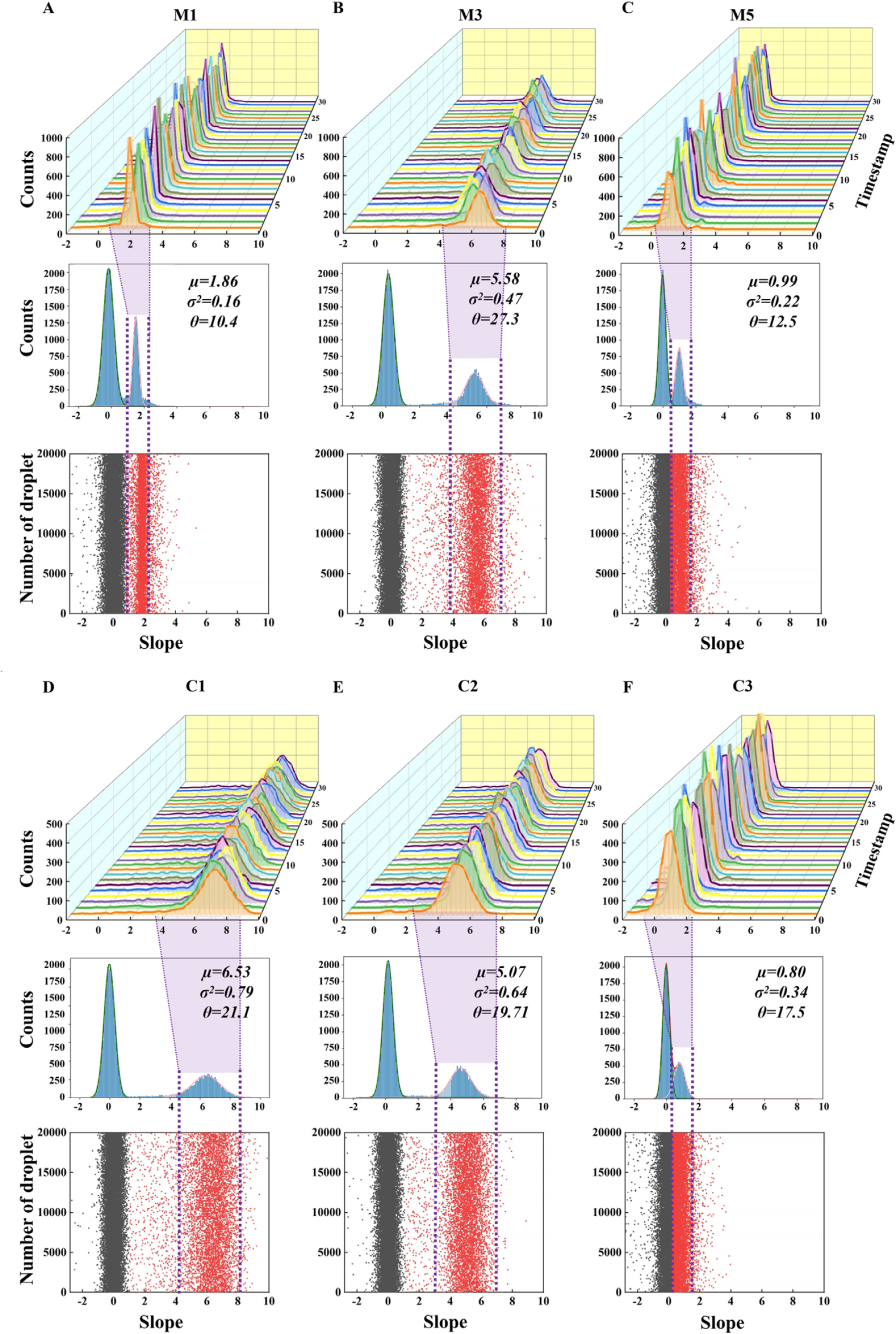
Comparative analysis of multi‐activity characteristics for mutant and commercial phi29 DNA polymerases. The dSMAT platform was used to simultaneously quantify the catalytic synthesis rate (*µ*, expected value of slope distribution), heterogeneity (*σ^2^*, variance), and temporal synthesis variation (*θ*, coefficient of variation of *µ* over time) of three mutant (M1, M3, M5) and three commercial (C1, C2, C3) phi29 DNA polymerase variants at the single‐molecule level. Each panel presents a triad of analyses for one enzyme. (A–C) Characterization of mutant polymerase. (D–F) Characterization of commercial polymerase. For each enzyme (A–F): Top row: The statistical distribution of the catalytic slope (*k*) across all positive microwells evolves over the 900‐min experiment. The solid line tracks the temporal trajectory of the mean value (*µ_n_*) for each time interval. Middle row: The frequency distribution of the final calibrated slope values (*k*) from all single‐molecule reactions. The curve represents a Gaussian fit to the distribution, the center of which defines *µ*, and the variance defines *σ^2^*. Bottom row: A scatter plot of every individual slope value obtained, illustrating the dispersion and clustering of single‐molecule activities. Each point represents the catalytic rate of a single enzyme molecule. The data highlight stark disparities in reaction kinetics among polymerase variants. While M5 prioritizes uniformity (low *σ^2^*) and processive stability (low *θ*), commercial variant C1 exhibits the highest basal rate but severely lacks intermolecular uniformity (*n* > 1000 active wells per condition).

Leveraging the high‐throughput capability of dSMAT, we characterized the kinetic landscapes of three engineered mutants (M1, M3, M5) and three commercial variants (C1, C2, C3) to identify optimal candidates for sequencing (Figure 5). The comparative analysis reveals a critical trade‐off between polymerization velocity and population uniformity that is often obscured in bulk assays. Among the engineered variants, mutant M3 exhibited a high polymerization velocity (*µ* = 5.58) but exhibited significant instability (*θ* = 27.3%) and intermolecular heterogeneity (*σ^2^* = 0.47). In stark contrast, mutant M5 displayed a kinetically restrained yet exceptionally consistent profile: despite a moderate polymerization velocity (*µ* = 0.99), it achieved the lowest population heterogeneity (*σ^2^* = 0.22) and temporal variation (*θ* = 12.5%) among all tested variants.

Parallel profiling of commercial preparations reveals similar functional divergence. For instance, variant C1 exhibited higher polymerization velocity (*µ* = 6.53) concurrent with higher population heterogeneity (*σ^2^* = 0.79), whereas C3 resembled the high‐fidelity profile of M5 (*µ* = 0.80, *σ^2^* = 0.34). These data demonstrate the capacity of dSMAT to decouple intrinsic catalytic rate from functional stability. By identifying M5 as the variant with the most consistent molecular performance, we establish that optimizing for low *θ* (stability) and low *σ^2^* (heterogeneity)—rather than turnover velocity—is the key strategy for enhancing long‐read sequencing fidelity [37].

### 2.5 Effect of Laser Physical Treatment of Different phi29 DNA Polymerases

To distinguish intrinsic photochemical instability from macroscopic thermal artifacts, enzyme activity was profiled under strict active thermal clamping. Enzyme aliquots (10 µL) were subjected to high‐intensity laser irradiation (30 mW, 532 nm) while immersed in an ice‐water bath (∼4°C), serving as a robust thermal sink. Thermodynamic modeling confirms that steady‐state heating remained negligible (< 0.1 K) due to the efficient heat dissipation and minimal optical absorption of the aqueous buffer (Figure S7). This experimental design ensures that the observed functional degradation is predominantly driven by discrete photochemical modifications rather than bulk thermal unfolding. While the optical geometry of focused irradiation in dSMAT differs from the evanescent field confinement of ZMWs, our protocol exposes the polymerase to a comparable integrated photon flux density. To bridge the gap between laboratory optical stress tests and actual sequencing runtimes, an accelerated photochemical aging (APA) model was developed (Table S6). This design effectively functions as a physiological surrogate, replicating the cumulative oxidative stress characteristic of long‐read sequencing workflows while enabling the precise decoupling of photochemical damage from thermal effects.

**FIGURE 6:**
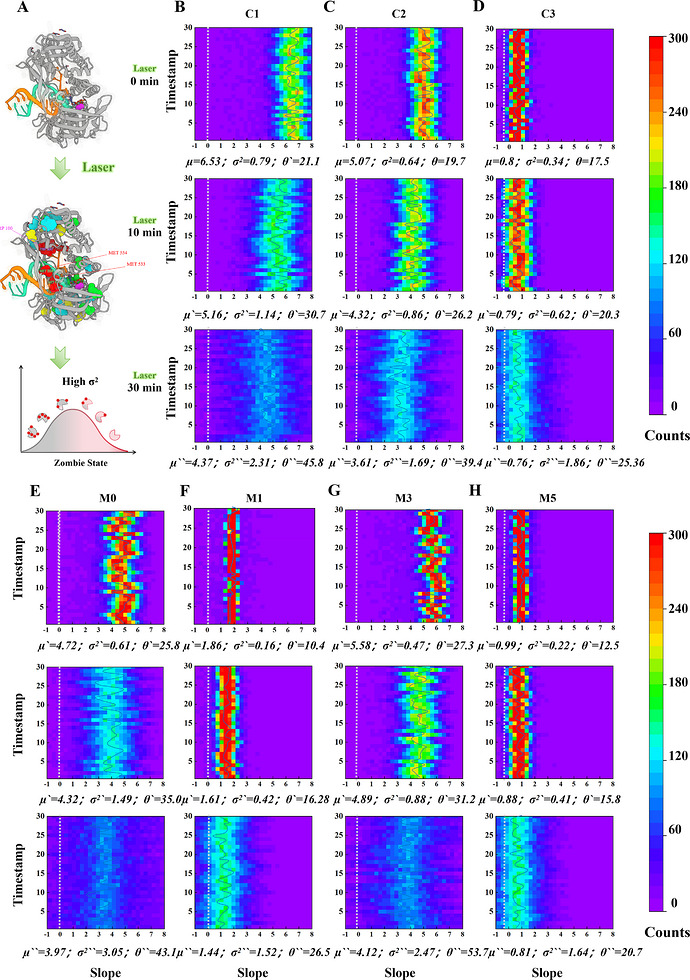
Evolution of laser‐induced oxidative scarring and kinetic heterogeneity in phi29 DNA polymerases. (A) Mechanistic model of oxidative scarring. Fundamentally different from thermal denaturation, which involves a binary all‐or‐none transition, high‐intensity laser irradiation induces the cumulative accumulation of oxidative modifications on interface‐proximal residues within the aqueous translocation pathway [45]. As exposure time increases (from 0 to 30 min), this stochastic damage likely leads to increased steric friction, transforming the native homogeneous population into a broad, kinetically impaired state (high *σ^2^*, indicated as the “Zombie State”). (B–D) Time‐resolved single‐molecule kinetic profiling of commercial polymerase variants (C1, C2, and C3). (E–H) Time‐resolved kinetic profiling of wild‐type and engineered mutants (M0, M1, M3, and M5) (*n* > 1000 active wells per condition). Heatmaps display the distribution of catalytic rates (Slope) across the population over time (Y‐axis: Timestamp within the assay). The progression from top to bottom in each column (0 min to 30 min laser exposure) reveals the gradual broadening of the kinetic spectrum. Quantitative parameters—catalytic rate (*µ*), heterogeneity (*σ^2^*), and temporal synthesis variation (*θ*)—are annotated below each panel. Notably, wild‐type M0 and C1 exhibit severe oxidative scarring characterized by a drastic increase in *σ^2^* and loss of stability (*θ*) after 30 min. In contrast, mutant M5 showed greater resistance, remained relatively tightly distributed (*σ^2^”* = 1.64), and highest stability (*θ”* = 20.7%) post‐irradiation, confirming its suitability for long‐read optical sequencing.

Different from the uniform binary transition typical of thermal denaturation, laser stress instead drove a progressive kinetic broadening. As visualized in the time‐resolved heatmaps (Figure 6B–H), the initially tight rate distributions of native enzymes flattened into broad, heterogeneous spectra. The wild‐type M0 exhibited a significant increase in kinetic heterogeneity (*σ^2^* increased from 0.61 to 3.05) accompanied by a decline in synthesis rate (*µ*). This continuous spectrum indicates the generation of diverse kinetically impaired subpopulations characterized by reduced rates and increased instability.

Comparative profiling reveals significant differences in resilience among variants. Commercial enzymes proved highly susceptible to photo‐oxidative stress, exhibiting severe broadening and stability loss. In contrast, the engineered mutants M1 and M5 demonstrated enhanced robustness, maintaining lower kinetic heterogeneity and temporal variation post‐irradiation. Notably, M5 may have emerged as the most photostable candidate for optical sequencing applications.

We attribute this laser‐induced kinetic broadening to a mechanism of “oxidative scarring.” Structural mapping of the phi29 ternary complex (PDB: 2PYL) reveals a dense cluster of solvent‐accessible, electron‐rich residues—specifically, Methionine, Tryptophan, and Cysteine—flanking the DNA translocation channel (Figure 6A). Under laser irradiation, we propose that ROS stochastically modify these residues, creating random “molecular scars.” In contrast to cooperative denaturation, these discrete chemical modifications introduce localized steric heterogeneity and friction without disrupting the global fold [44]. Since each molecule accumulates a random pattern of oxidative events, the population manifests as a widened variance in catalytic rates (*µ*), effectively creating a library of active‐but‐impaired variants detrimental to sequencing accuracy.

To interpret the biophysical origin of the progressive broadening of the kinetic spectrum (*σ^2^*), we performed comprehensive structural mapping based on the phi29 ternary complex (PDB: 2PYL; Figure S8). Structural modeling revealed that while global thermal unfolding was suppressed via active clamping, high‐irradiance exposure selectively targets redox‐sensitive motifs (Met, Trp, and Cys). Notably, it suggested that residues lining the translocation channel, such as Met533 and Trp100 (positioned within a 5.0 Å radius of the DNA translocation cleft), are vulnerable targets for ROS attack due to their functional accessibility (Figure S8C–E). The conversion of these residues into bulky polar derivatives induces quantized steric impediments—designated as oxidative scars—along the polymerase‐DNA interface. This stochastic roughening of the conformational landscape effectively elevates activation barriers for sliding coordination, thereby transforming the native homogeneous enzyme population into a diverse spectrum of kinetically impaired sub‐states.

Beyond quantifying the attenuation of activity, we further investigated the potential mechanism behind the laser‐induced effects observed in Figure 6. Our results demonstrate that laser irradiation induces a quantifiable alteration in the activity metrics (*µ, σ^2^, θ*) of phi29 DNA polymerases. To advance beyond a phenomenological description of laser‐induced inhibition, we next investigated the underlying biochemical mechanism.

### 2.6 Chemical Verification of the Oxidative Scarring Mechanism

**FIGURE 7:**
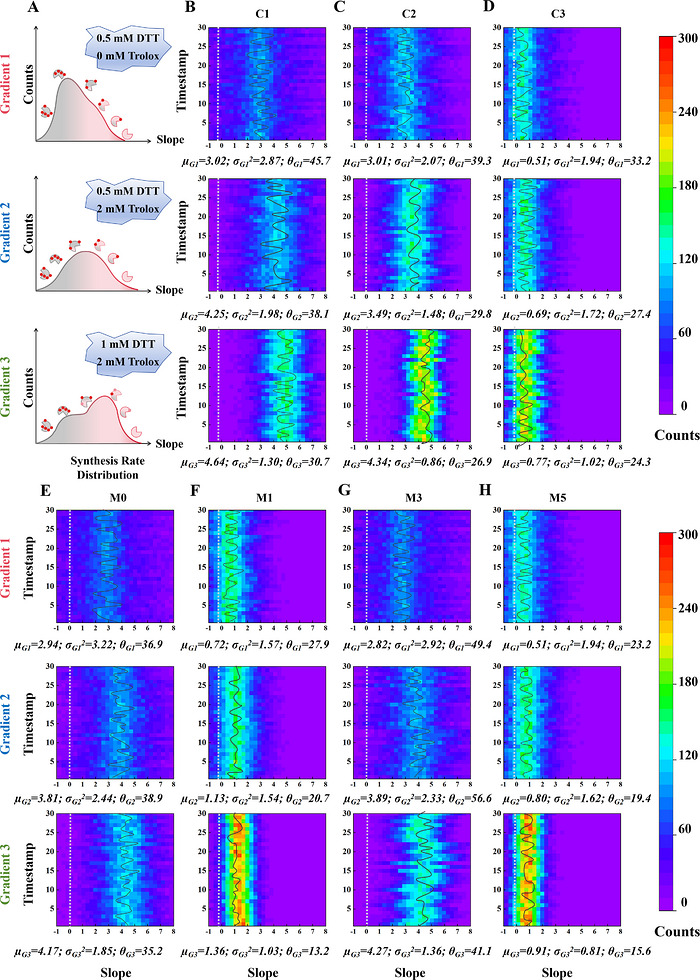
Dissecting the mechanism of oxidative scarring via chemical rescue and buffer optimization. (A) Schematic illustration of the catalytic rate distributions under distinct reductive‐antioxidant environments. Top (Gradient 1): In the absence of a specific quencher (0 mm Trolox), photo‐oxidation of DTT generates ROS, creating a heterogeneous oxidatively scarred population with a broadened rate spectrum. Middle (Gradient 2): Buffer containing Trolox (2 mm) specifically quenches ROS, narrowing the distribution and rescuing activity despite low reductant levels. Bottom (Gradient 3): Synergistic optimization (DTT + Trolox) restores the native homogeneous state. (B–H) Real‐time single‐molecule kinetic profiling of three commercial (C1–C3) and four engineered (M0, M1, M3, M5) phi29 DNA polymerases under high‐intensity laser stress (30 mW, 30 min, 532 nm). Heatmaps display the temporal evolution of catalytic rates (slope) across three buffer formulations.

To reveal this photochemical mechanism, we performed a biochemical rescue experiment using ROS scavengers (Figure 7). The presence of an antioxidant (Trolox) during irradiation successfully prevented the decline in catalytic rate (*µ*) and the broadening of kinetic rate distributions (*σ^2^*). Since scavengers neutralize oxidative radicals but do not affect thermal dissipation, this result provides functional evidence that the laser‐induced functional decline is driven by oxidative modification of the enzyme. These kinetically impaired, yet continuously processive subpopulations are particularly detrimental in sequencing applications as they desynchronize signal readout [46], a critical kinetic nuance that our dSMAT methodology successfully captures, which is inherently masked in traditional ensemble biochemical assays.

We performed a controlled titration experiment under fixed laser stress (30 mW, 30 min) and analyzed the performance of polymerase variants in three distinct buffer formulations designed to probe the interplay between Dithiothreitol (DTT) and Trolox (Figure 7). The analysis of buffer gradients reveals a critical trade‐off involving DTT. Paradoxically, our buffer titration unmasked conventional DTT as an unexpected phototoxicity catalyst under specific laser exposure, where DTT essentially serves as a sacrificial electron donor feeding ROS generation. In baseline deficiency (Gradient 1) of C1, reducing the DTT concentration (0.5 mM, without Trolox) resulted in severely compromised activity (*µ_G1_* dropped to 3.02). This impairment arose not merely from insufficient reducing power, but rather because the enzyme remained continuously exposed to ROS generated by the DTT‐driven photo‐oxidative cycle. Crucially, the specific rescue (Gradient 2) experiment—where adding 2.0 mM Trolox to the 0.5 mM DTT buffer restored activity—provides strong support. This result corroborates that Trolox specifically quenches singlet oxygen and superoxide radicals photochemically generated from trace impurities [47], functionally uncoupling reduction maintenance from ROS mitigation. Finally, synergistic optimization (Gradient 3), which combined standard DTT (1.0 mM) with Trolox (2.0 mM), achieved the highest stability (*σ_G3_^2^* = 0.81, M5). This result demonstrates that the optimal strategy requires a dual approach—DTT to maintain a reductive environment for Cysteines, and Trolox to specifically scavenge the ROS byproducts generated in the photo‐oxidative microenvironment. This reduction‐antioxidant synergy decouples the conflicting roles of the reaction buffer components [48].

The biochemical rescue experiment demonstrates that the optimized reductive‐antioxidant buffer formulation significantly restores enzymatic heterogeneity on the chip. However, a crucial question remains: do these microfluidic kinetic fingerprints accurately predict performance in a real sequencing setup? To bridge the gap between our in vitro dSMAT metrics and actual genomic data, we performed a cross‐platform validation using SMRT sequencing.

### 2.7 Quantitative Verification of Kinetic Parameters via SMRT Sequencing

To validate the predictive power of dSMAT, we performed SMRT sequencing using the commercial polymerase C3. By analyzing the Local Base Rate (Data S6), we establish a comprehensive quantitative correlation across all three dSMAT‐derived kinetic dimensions (*µ, σ^2^, θ*).

**FIGURE 8:**
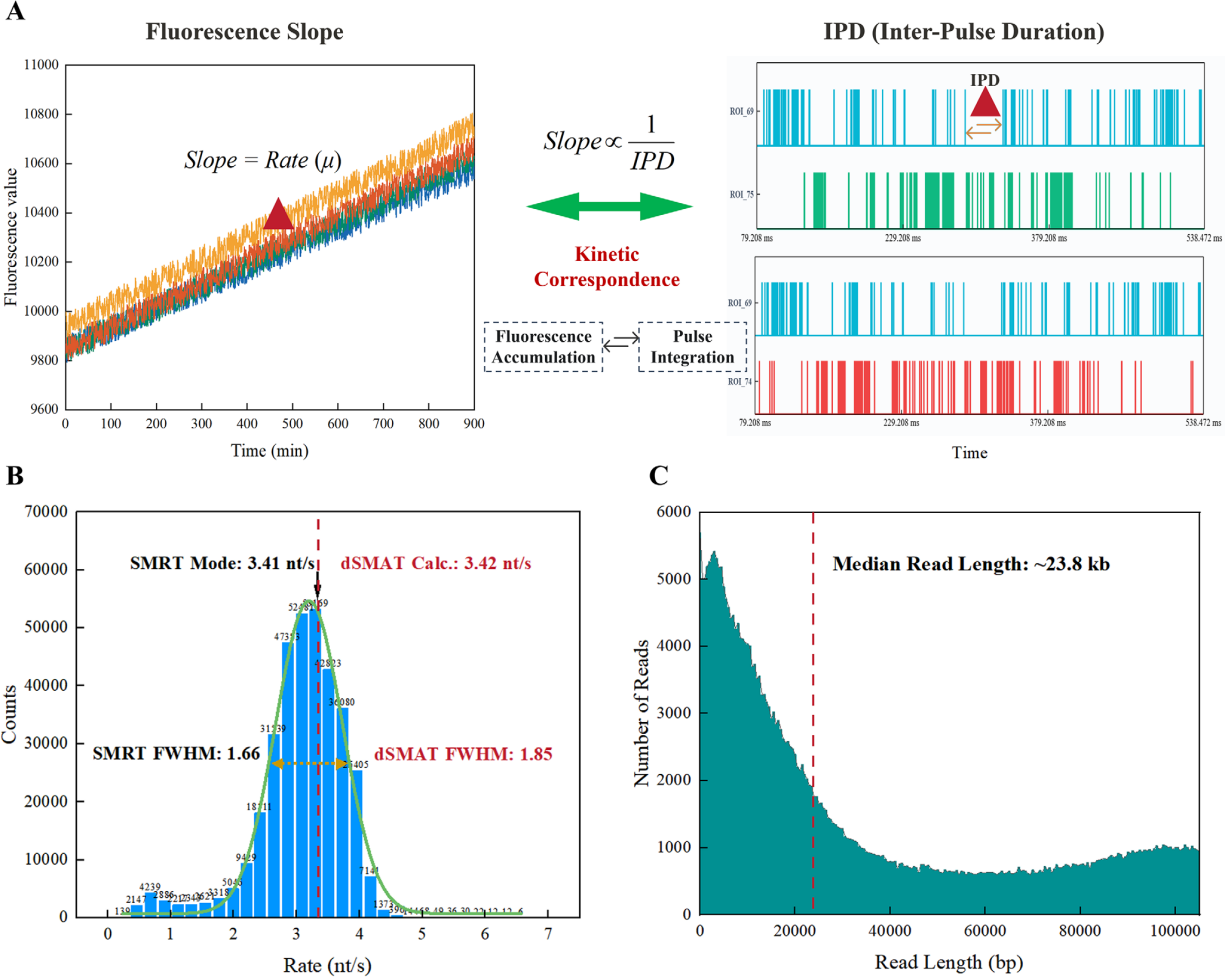
Cross‐platform kinetic validation using SMRT sequencing data. (A) Schematic of signal correspondence. The continuous fluorescence accumulation observed in dSMAT (left, fluorescence accumulation trace) represents the integration of discrete nucleotide incorporation pulses (IPD) captured in SMRT sequencing (right, pulse trace). The fluorescence slope is mechanistically linked to the pulse frequency (1/IPD). (B) Quantitative validation of catalytic rate and heterogeneity (compare the SMRT sequencing and a continuous 10‐min dSMAT treatment under 10X objective). The histogram displays the distribution of Local Base Rates extracted from SMRT sequencing. The distribution exhibits a mode at 3.41 nt/s (black arrow), which is nearly identical to the dSMAT‐calculated absolute rate of 3.42 nt/s (red dashed line). Furthermore, the kinetic heterogeneity is quantitatively validated: the FWHM of the sequencing distribution is 1.66, closely matching the theoretical FWHM of 1.85 derived from the dSMAT heterogeneity parameter (*σ^2^* = 0.62). (C) Read length distribution of C3 polymerase. The histogram shows a median read length of approximately 23.8 kb (dashed line) with a substantial tail extending beyond 100 kb. This long‐range processivity validated the temporal synthesis variation parameter (*θ*) characterized via the dSMAT platform.

First, we robustly establish the physical link between the two platforms. In dSMAT, the enzymatic activity is recorded as a continuous fluorescence accumulation. As illustrated in Figure 8A, this macroscopic slope mathematically represents the integration of discrete nucleotide incorporation events. The steepness of the fluorescence slope is inversely proportional to the Inter‐Pulse Duration (IPD) observed in SMRT traces [7].

Based on the accelerated photo‐aging model, the kinetic profile of C3 polymerase after 10 min of stress (equivalent time = 3.33 h) was benchmarked against the SMRT “Local Base Rate” dataset. This calibration point accounts for the cumulative workload of a standard 10‐h sequencing session, an effective excitation duty cycle of 31.2% as derived from pulse‐trace analytics (Data S6; equivalent cumulative load = 3.12 h). We observed a remarkably high peak‐to‐peak concordance: the predicted velocity from dSMAT (3.42 nt/s) and the operational SMRT mode (3.41 nt/s) displayed an equivalent error of less than 1.0%. This alignment demonstrates that dSMAT can reconstruct the biophysical signature of an active motor protein under realistic machine load.

The SMRT sequencing data reveals a rate distribution with a dominant peak mode at 3.41 nt/s (Figure 8B). By introducing a specific analytical volumetric conversion formula (refer to Equation (3) and Supplementary Text and Equation (S2) defining *r_ca_*), we bridged these two conceptual spaces. Mathematically, the unit‐less population expected activity peak—originally extracted on the dSMAT chip as a relative expected signal accumulation value (*µ* = 0.79 RFU ∙ min^−1^, formally defining our relative mean catalytic capability)—was reliably deciphered into an absolute nucleotide extension velocity (*r_ca_* = 3.42 nt/s). The close agreement (equivalent error < 1%) confirms that dSMAT accurately quantifies the mean physiological kinetics. Crucially, our dSMAT algorithm filters for high‐linearity fluorescence trajectories (R^2^ > 0.90) to exclude stalled states. Consequently, the dSMAT‐derived rate (3.42 nt/s) specifically mirrors the “Local Base Rate” mode (3.41 nt/s) in SMRT sequencing, an established parameter reflecting the true active polymerization velocity. This mechanistic alignment confirms that dSMAT accurately extracts the steady‐state active polymerization velocity, decoupling it from off‐pathway stalling events.

To validate the heterogeneity parameter *σ^2^*, we quantified the kinetic dispersion. In dSMAT, C3 exhibits a variance of *σ^2^* = 0.62, which theoretically corresponds to a full width at half maximum (FWHM) of 1.85 (calculated as Equation (5)). Gaussian fitting of the SMRT sequencing rate distribution yielded an experimental FWHM of 1.66 (Figure 8B). The high concordance between the predicted and observed peak widths confirms that the *σ^2^* parameter in dSMAT is not a fitting artifact but a physical measure of the intrinsic inter‐molecular heterogeneity of the enzyme population [49].

Finally, we validated the temporal synthesis variation parameter *θ*. Theoretically, for a stochastic enzyme, the temporal variation (*θ*) should correlate with the population CV. The CV derived from SMRT data (StdDev/Mean = 22.1%) closely aligns with the stability coefficient measured on dSMAT (10‐min treatment, *θ* = 20.3%). This stability metric successfully predicted the functional outcome: C3 generated a robust read length distribution with a median of approximately 23.8 kb and a long tail extending beyond 100 kb (Figure 8C).

The numerical convergence of rate (*µ*), dispersion (*σ^2^*), and stability (*θ*) between the microfluidic chip and the sequencer proves that dSMAT captures the full kinetic fingerprint of DNA polymerases, enabling high‐fidelity screening prior to sequencing.

## 3 Discussion

### 3.1 Advantages of the Digitization Chip Approach

The dSMAT platform offers distinct advantages over traditional bulk methods for enzyme activity assessment. Foremost, digitization into 432 pL volumes alleviates substrate depletion—a pervasive limitation in single‐molecule enzymology. In contrast to the spatial constraints of femtoliter ZMWs, our chambers function as stable thermodynamic reservoirs, ensuring that the extracted heterogeneity (*σ^2^*) accurately reflects intrinsic conformational memory.

The platform ensures the compartmentalization of individual enzymatic reactions, eliminating intermolecular interference and enabling the detection of rare stochastic events often masked in bulk measurements. Moreover, the high‐throughput nature of the microfluidic array allows for the simultaneous analysis of thousands of complexes, providing high statistical power that is often limited in conventional single‐molecule techniques. This scale is particularly valuable for screening large enzyme libraries or ensuring data reliability through massive replication. Finally, building upon real‐time fluorescence monitoring and quantitative kinetics in microwell arrays [50, 51], the dynamic readout quantifies multi‐feature parameters for individual enzymes independently, providing absolute activity measurements. In addition, the platform serves as a robust functional predictor, insensitive to minor volumetric tolerances in microfluidic fabrication. In contrast to closed‐architecture commercial ZMW platforms, dSMAT offers an open system compatible with custom buffer formulations and modified substrates, thereby democratizing the screening of processive enzymes.

### 3.2 Optimization of Data Processing Algorithms

The high consistency and narrow bootstrap confidence intervals of the kinetic parameters (*µ, σ^2^, θ*) in independent biological replicate experiments (Table S1) demonstrate the validity and reproducibility of the statistical results of the dSMAT platform. These results also validate the effectiveness of the dSMAT‐GMM (Gaussian Mixture Model) framework as an analysis for profiling functional heterogeneity. By bypassing the limitations of ensemble methods, this approach simultaneously resolves distinct subpopulations and extracts their kinetic parameters, including catalytic synthesis rate (*µ_i_*), inter‐molecular kinetic heterogeneity (*σ_i_^2^*), and temporal continuous synthesis stability (*θ*_i_). The parameter *σ_i_^2^* reflects the dynamic heterogeneity within these subpopulations, which may originate from intrinsic conformational fluctuations (Figure S6 and Table S5). The strong correlation between different clusters in the scatter plot further confirms this conjecture. While pioneering single‐molecule studies have long established that individual enzyme catalysts natively undergo stochastic functional fluctuations—cycling through transient active and paused states—our dSMAT framework extends this perspective by uncoupling innate fluctuations from non‐equilibrium damage constraints. This reveals that enzyme catalysis is a highly dynamic, non‐equilibrium process occurring in complex biochemical environments. These fluctuations reflect the coupling of dynamic reaction conditions, intrinsic conformational behaviors of individual enzyme molecules, and stochastic microscopic events. By capturing these sophisticated regulatory mechanisms and reaction characteristics—which are inherently masked in bulk ensemble assays—dSMAT provides a fundamentally more accurate depiction of single‐molecule behavior. The strong correlation between computationally assigned components and distinct clusters in scatter plots confirms the algorithm's accuracy. This methodology enables the precise quantitative characterization of enzymatic systems, applicable to kinetics, heterogeneous, and mutation studies at the single‐molecule level. Future work should focus on refining algorithms to further improve positive chamber identification and parameter extraction. Machine learning techniques, such as neural networks or support vector machines, could be employed to enhance pattern recognition and adapt to data variability [52, 53]. Moreover, integrating advanced statistical models like Bayesian inference or Hidden Markov Models (HMM) would provide a more nuanced understanding of underlying kinetic mechanisms. These models will assist in deciphering complex stochastic behaviors and predicting enzyme performance under diverse physiological conditions.

### 3.3 Mechanistic Insights into Laser‐Induced Functional Heterogeneity

We identify a critical biophysical distinction between the photo‐oxidative mechanism characterized here and classical thermal denaturation. Whereas thermal stress induces a cooperative, global unfolding transition that results in binary inactivation (an all‐or‐none state [54]; Figure S10), photo‐oxidative stress manifests as a progressive accumulation of kinetic barriers. We demonstrate that the discrete oxidation of interface‐exposed residues does not immediately disrupt the global fold but instead cumulatively stiffens the enzyme's conformational landscape. This creates a spectrum of kinetically impaired subpopulations characterized by increased internal friction and stochastic stalling. Consequently, the primary determinant of read length in optical sequencing is not necessarily complete structural unfolding, but rather the gradual accrual of oxidative modifications that render the polymerase's turnover frequency kinetically incompatible with the continuous signal acquisition requirements of the sequencer. dSMAT is highly effective at resolving this desynchronization, which appears macroscopically as increased kinetic dispersion (*σ^2^*) rather than immediate signal loss.

Because conventional ensemble methods report population‐averaged metrics, they inherently mask microscopic kinetic diversity. In contrast, single‐molecule resolution reveals that laser irradiation expands the tightly defined native rate distribution into a broad, dispersed kinetic spectrum (high *σ^2^*, Figure 6). This dispersion is mechanistically grounded in the structural topology of the phi29 ternary complex (Figure 6A), where the DNA translocation tunnel and template‐binding cleft are lined with oxidation‐sensitive residues (Figure S8). Structural mapping of the phi29 ternary complex reveals that although the protein core remains shielded, the sensitive residues—e.g., Met533, Trp100, and Cys530—are strategically lining the DNA translocation channel and the template‐binding cleft. These residues are functionally accessible to photo‐activated ROS diffused through the solvent‐filled catalytic pathways. We propose that the oxidative scarring originates from the localized conversion of these hydrophobic/thio‐aromatic side chains into bulky polar adducts, thereby increasing potential molecular friction and activating conformational barriers precisely where the motor‐DNA interaction occurs. While traditional bulk mass spectrometry could identify the presence of oxidized residues, linking specific chemical modifications to their corresponding functional kinetic sub‐states remains a profound challenge due to the stochastic, single‐molecule nature of these events. Therefore, we present an oxidative scarring model supported by chemical rescue and structural exclusion, which demonstrates the kinetic dispersion (*σ^2^*) that ensemble methods inherently mask.

Distinct from global denaturation, these random localized perturbations introduce steric impediments and alter electrostatic potentials along the translocation path [55]. Because the number and spatial distribution of these oxidative events vary stochastically among individual molecules, the population exhibits a widened variance in catalytic rates (*σ^2^*). This aligns with biophysical models where Methionine oxidation increases the roughness of the energy landscape, elevating activation barriers for key conformational transitions, thereby precipitating the observed increase in off‐pathway pause events [56]. Importantly, because most processive DNA polymerases (including proprietary variants used in commercial long‐read sequencing) share highly conserved topological structures and universally rely on similar redox‐sensitive residues (Met, Trp, Cys) lining their DNA translocation channels, we speculate that this oxidative scarring mechanism represents a generalized failure mode for diverse motor proteins operating under high‐photon‐flux conditions.

### 3.4 Decoupling Photochemical Toxicity from Buffer Chemistry

The biochemical rescue experiments provide strong biochemical support for distinguishing photo‐oxidative impairment from macroscopic thermal inactivation. Crucially, these results resolve a pervasive confounding factor in single‐molecule enzymology: the role of DTT. While standardly employed as a reductant for catalytic cysteines, DTT unmasks a buffer chemistry paradox under high photon flux, where it displays strong support for acting as a sacrificial electron donor that promotes the local ROS reservoir under specific green laser saturation. Mechanistically, under continuous high‐photon‐flux laser irradiation and in the presence of trace transition metals, DTT undergoes accelerated auto‐oxidation. Rather than merely acting as a reductant, it acts as a sacrificial electron donor that continuously generates thiyl radicals and superoxide anions. Without a specific radical scavenger to intercept these byproducts, DTT inadvertently sustains a local ROS reservoir, driving the oxidative scarring. The specific restoration of kinetic homogeneity (*σ^2^*) and catalytic efficiency (*µ*) by Trolox—which intercepts radicals without altering thermodynamic parameters—serves as an orthogonal verification that the observed functional decline is driven by cumulative chemical erosion rather than cooperative protein unfolding.

This finding necessitates a shift in enzymatic stabilization strategies: the Reduction‐Antioxidant Synergistic (RAS) paradigm. By decoupling the requirement for chemical reduction from immediate photochemical radical interception, the RAS paradigm provides a high‐fidelity roadmap for maximizing the read‐length limits of sequencing motors. This study corroborates that maintaining long‐term enzymatic stability depends as much on the non‐equilibrium chemical environment as on the intrinsic robustness of the protein scaffold. The dSMAT platform lays the groundwork for comprehensive multidimensional buffer engineering. For example, exploring saturation gradients of Trolox or screening alternative quenchers like Ascorbic Acid or L‐Histidine could further maximize antioxidant protection. Beyond DNA polymerases, the RAS framework is broadly applicable to diverse light‐intensive systems, including optical trapping and single‐molecule fluorescence assays, where preserving kinetic consistency is critical for high‐resolution molecular analysis.

### 3.5 Future Directions

This study lays the foundation for applying the digital chip approach to enzyme activity assessment; specifically, the dSMAT architecture offers a generalized framework for characterizing molecular motors under non‐equilibrium conditions. By replacing the specific molecular beacon, this platform can be readily adapted to profile the photo‐stability of other optically‐dependent biological systems, such as helicase stepping mechanics under optical trapping‐like intensities, or the kinetics of light‐activated enzymes (optogenetics) where distinguishing functional activation from photodamage is critical. Moreover, the continued development of user‐friendly data analysis software and standardized protocols will facilitate the broader adoption of this technology in research and industrial settings. Advancements in microfluidics, optics, and computational methods will further enhance the capabilities of the dSMAT strategy, establishing it as a valuable platform for enzyme research. Furthermore, integrating dSMAT with computational models of enzyme kinetics could provide a comprehensive understanding of enzyme behavior, facilitating the rational design of enzymes with tailored properties for specific applications.

The standard system utilized in our dSMAT strategy has demonstrated robust capabilities in assessing the functional heterogeneity of individual polymerases (Figure S1C). However, while cross‐platform single‐molecule data (dSMAT and SMRT) physically corroborate the functional consequences of oxidative scarring, achieving the exact chemical stoichiometry of this mechanism remains a future milestone. Bridging bulk proteomic mapping with single‐molecule kinetic heterogeneity remains a profound frontier. Meanwhile, there is potential for further refinement. Reaction parameters—including temperature, pH, and substrate concentration—can be fine‐tuned to maximize assay sensitivity and specificity. For instance, conducting experiments across a temperature gradient could elucidate the thermal stability of enzymes and determine optimal operating conditions.

Additionally, the microfluidic chip design could be improved to minimize sample coalescence during injection, a critical factor for accurate single‐molecule analysis. More importantly, while DTT exhibits radical‐forming potential under specific high‐flux contexts, this represents a unique non‐equilibrium edge case for current long‐read buffers. Future studies could evaluate the substitution of DTT with alternative non‐thiol reductants, such as Tris(2‐carboxyethyl)phosphine (TCEP). Combining machine learning with the high‐throughput capability of dSMAT may address this complex multidimensional buffer engineering challenge. Future innovations, such as the incorporation of anti‐adhesive coatings or dynamic flow control modules, would further stabilize fluidics and reduce injection variability.

In the future, the dSMAT strategy can evolve into a general‐purpose platform with transformative potential across multiple fields of enzyme research. Broadly applicable beyond SMRT sequencing polymerases, its capability for high‐throughput, single‐molecule analysis makes it ideally suited for enzyme engineering, enabling the screening of vast mutant libraries to identify variants with optimized multi‐activity traits, such as enhanced fidelity or precisely modulated nuclease activity.

## 4 Conclusion

In summary, the dSMAT strategy represents a paradigm shift from static ensemble averaging to dynamic single‐molecule enzymology, resolving the long‐standing challenge of profiling kinetic heterogeneity under physiological sequencing conditions. Unlike conventional assays that mask stochastic behaviors, our platform successfully decouples the intrinsic catalytic rate (*µ*), intermolecular heterogeneity (*σ^2^*), and continuous synthesis stability (*θ*) at a high‐throughput scale.

Crucially, this multidimensional analysis unveils a previously overlooked failure mode in sequencing enzymes: photo‐oxidative scarring. We demonstrated that laser irradiation induces a stochastic broadening of the kinetic spectrum rather than simple thermal denaturation, a kinetic mechanism strongly corroborated by our ROS‐scavenging rescue experiments and cross‐platform SMRT sequencing validations. Our findings highlight a critical photochemical paradox in standard enzyme storage buffers: reducing agents like DTT can unintentionally fuel the generation of ROS. Therefore, we propose that a synergistic reductive‐antioxidant formulation is a prerequisite for preserving single‐molecule kinetics under high‐intensity illumination. Furthermore, we established the robust predictive power of dSMAT by demonstrating a precise quantitative link between microfluidic kinetic fingerprints and SMRT sequencing metrics, specifically mapping the stability parameter (*θ*) to sequencing read length and the heterogeneity index (*σ^2^*) to *IPD* variance.

By bridging the gap between protein engineering and genomic applications, dSMAT provides a transformative framework for the rational design of next‐generation enzymes. We envision that this platform will not only accelerate the screening of photostable polymerases for ultra‐long read sequencing but also broaden its applicability to fundamental mechanistic studies, diagnostic assay development, and the precision evolution of biocatalysts for complex synthetic biology applications.

## 5 Materials and Methods

### 5.1 Design and Preparation of Molecular Beacons and Template Sequences

The reaction system was optimized to contain 50 mm Tris‐HCl (pH 7.5), 10 mm MgCl_2_, 100 mm KCl, 1 mm DTT, 0.1 mg/mL BSA, and 5 µm molecular beacon. The molecular beacon features a 35‐nt stem‐loop structure (Table S2) with a Melting Temperature (*T_m_*) of 72.16°C. In its closed state, the fluorophore is quenched; hybridization to the RCA product separates the fluorophore from the quencher, emitting fluorescence. As depicted in Figure 2A, a single‐stranded DNA template was circularized using T4 DNA ligase and incubated with phi29 DNA polymerase and a thiol‐modified primer at 4°C for 20 min to form a stable enzyme‐primer‐template ternary complex [57]. RCA was initiated by adding dNTPs. Beacon hybridization (*τ* ≈ 0.2 s) is orders of magnitude more rapid than enzymatic synthesis (*τ* > 18 s), ensuring that fluorescence accumulation is predominantly rate‐limited by the polymerization step.

### 5.2 Principles and Calculation of Kinetic Parameters

The core derived parameters that characterize the macro‐biological profile matrices are defined as follows:

The global mean catalytic rate *µ* represents the population‐averaged intrinsic catalytic capability obtained from Gaussian mixture model clustering. This global parameter corresponds to the central expectation of catalytic activities across all valid traces and serves as the fundamental steady‐state metric of the system.

The time‐dependent local catalytic rate *µ_t_* denotes the instantaneous catalytic activity within a defined temporal observation window. This time‐varying quantity reflects the local dynamic behavior of individual trajectories at each time interval.

The intrinsic heterogeneity parameter *σ^2^* describes the population dispersion and spatial‐functional heterogeneity of catalytic activities. It is defined as the global variance derived from the Gaussian distribution of catalytic rates, representing the magnitude of intrinsic differences among individual molecular trajectories.

The temporal fluctuation index *θ* quantifies the dynamic stability and processivity variation of catalytic activity. It is calculated as the normalized coefficient of variation of the time‐dependent local rates *µ_t_*, characterizing the degree of temporal instability and kinetic fluctuations along the entire reaction trajectory. Physically, the parameter *θ* reflects the underlying conformational dynamics of the single enzyme. Because polymerases traverse a complex energy landscape, they inherently cycle between active synthesis and transient paused states. A low *θ* indicates a highly stable processivity with minimal entrapment in deep, non‐productive conformational states, which is a physical prerequisite for sustained, long‐read sequencing outputs.

To mitigate stochastic photon noise, raw fluorescence trajectories underwent a two‐stage smoothing process: intensity binning over a 6‐min window and slope derivation using a moving regression window of 5 points (∼30 min). Slope calibration (*k_raw_* to *k_corr_*) corrected for background and system response (Supporting Information).

Core kinetic parameters were calculated as follows:

(1) $$F\left(k_{\mathrm{corr}}\right)=\frac{1}{\sqrt{2\pi \sigma }}e^{-\frac{\left(k_{\mathrm{corr}}-\mu \right)}{2\sigma^{2}}}$$

(2) $$\theta_{m,n}=\frac{\sqrt{\frac{1}{N}\sum_{t=m}^{n}{\left(\mu_{t}-\mu_{\mathrm{mean}}\right)}^{2}}}{\mu_{\mathrm{mean}}}\times 100\%$$

The mathematical expectation (*µ*) of these single‐molecule k distributions effectively acts as a proportional metric to the overarching enzymatic catalytic velocity. Heterogeneity (*σ^2^*) is quantified as the variance of the *µ* distribution (*X ∼ N (µ, σ^2^)*). Temporal variation (*θ*) represents the coefficient of variation of *µ* over the 900‐min observation period. *N* defines the temporal sampling observation size window. Low *θ* values indicate high processivity crucial for long‐read sequencing.

### 5.3 Determination of Apparent Kinetic Efficiency Index and Cumulative Turnover Number

Conventional ensemble measurements mask stochastic fluctuations. To mathematically decouple the dynamic heterogeneity within these single‐molecule trajectories, we supplemented the basal rate (*µ*) and heterogeneity (*σ^2^*) with the apparent kinetic efficiency index (*K_app_*) and cumulative turnover number (*P*).

While the classical Michaelis constant (*K_m_*) is an equilibrium average, single‐molecule enzymes traverse a dynamic energy landscape. We derived *K_app_* as a real‐time probe of this kinetic state. Although catalytic turnover (*k_cat_*) and binding affinity (*K_d_*) are coupled, *K_app_* maps rate variations onto an equivalent substrate‐binding potential. This metric serves as a semi‐quantitative index of effective efficiency under non‐equilibrium conditions. Crucially, substrate consumption in the microwells is negligible (< 0.1%), validating the infinite reservoir assumption (constant [*S*]).

*K_app_* captures the enzyme's deviation from its theoretical maximum catalytic potential. Calculated via Equation (3):

(3) $$K_{\mathrm{app}}=\left[S\right]\left(\frac{V_{\max}}{\mu \left(t\right)}-1\right)$$

Here, V_max_ is the population‐averaged saturation rate determined from bulk kinetic titration, serving as a reference baseline [58]. An elevated *K_app_* indicates a conformational state with reduced catalytic efficiency (Figure S2).

While *µ* quantifies instantaneous speed, the cumulative turnover number (*P*) accounts for total productive capacity over the reaction duration.

*P* corresponds to the potential read length and is calculated as:

(4) $$P=\mu \cdot \phi_{\mathrm{vol}}\cdot T$$

where *Φ_vol_* is the volumetric conversion factor (260 molecules·pM^−1^ for 432 pL) and *T* is the reaction time (900 min). This metric mathematically acts as a macroscopic index, forecasting the expected continuous throughput capability of a specific polymerase under idealized continuous synthesis.

### 5.4 Design and Architecture of the Digitization Chip Platform

The dSMAT strategy leverages a high‐density silicon‐based microwell array as the core reaction substrate, designed to achieve robust digital partitioning. Fabricated via high‐precision microinjection molding, the chip features an array of approximately 20 000 independent micro‐reaction chambers etched into a polypropylene (PP) substrate. Each microwell (dimensions: 82 µm × 65 µm × 97 µm, 100 µm spacing) defines a precise reaction volume of 432 pL, ensuring high‐density integration (> 20 000 units/cm^2^) and suitable amplification product concentrations based on established digital assays [54]. The chip surface was modified to enhance hydrophilicity for uniform filling, while interstitial areas were rendered hydrophobic to ensure isolation and accurate quantification [55, 56]. This polymer‐based architecture offers excellent optical transparency, low auto‐fluorescence, and good thermal stability—critical factors for the precise temperature control and high signal‐to‐noise ratio (SNR) imaging required for single‐molecule kinetic monitoring. To establish the dSMAT platform, the chip was integrated with a custom fluorescence imaging system and sealed with an optical‐grade adhesive cover to prevent evaporation during long‐term (900 min) isothermal incubation.

### 5.5 Enzymes and Substrates

Wild‐type phi29 DNA polymerase, serving as the model enzyme, was obtained from commercial suppliers and stored under conditions maintaining activity and integrity. Circular DNA templates and hairpin probes were synthesized and HPLC‐purified to ensure high purity. Ligation reactions (20 µL) were assembled containing 10X T4 DNA ligase buffer, 2 µm primer DNA, and 0.5 µm template DNA in nuclease‐free water. The reaction was initiated by adding 2 µL of T4 DNA ligase (400 U/µL). The template‐primer mixture was annealed at 65°C for 5 min and cooled to 4°C for 2 min prior to enzyme addition. Ligation proceeded overnight at 16°C. Alternative incubation conditions (25°C for 2 h or 65°C for 10 min) were also tested. Following ligation, the mixture was treated with Exonuclease I and III at 37°C for 45 min to degrade unreacted ssDNA, followed by heat inactivation at 80°C for 15 min. Products were stored at 4°C or purified using commercial kits. Prior to dSMAT characterization, samples were prepared in the appropriate buffer (Table S4).

### 5.6 Fluorescence Detection System

Fluorescence signal acquisition was performed using a high‐sensitivity scientific CMOS camera. To ensure linear signal accumulation and minimize fluorophore photobleaching over the 900‐cycle experiment, optical parameters remained invariant throughout the duration of the assay. Images were captured at 1‐min intervals with an optimized exposure time (FAM: 100 ms; ROX: 300 ms), and an analog gain of 10 to utilize the camera's full dynamic range without triggering pixel saturation. This frequency sufficiently smooths high‐frequency photon shot noise while avoiding the severe fluorophore photobleaching that would occur at higher frame rates. The 1‐min sampling interval provides high‐fidelity tracking of enzymatic stability over the 15‐h duration without distorting the extracted kinetic metrics. The dSMAT platform was precisely maintained at 30°C to simulate physiological conditions. Images were systematically captured at 1‐min intervals over a 15 h, providing a high‐resolution record of enzymatic progression.

### 5.7 Statistical Analysis and Validation

#### 5.7.1 Data Acquisition and Analysis

The AI Mask Tracking algorithm was first employed to initialize the first frame, extracting the original mask list and using the “moments” function of OpenCV to obtain the centroid coordinates of the microwells. An iterative update strategy based on spatiotemporal consistency then mapped these masks to subsequent frames, dynamically tracking microwell masks and extracting the fluorescence value sequence through the “bitwise_and” function. Raw signals underwent normalization to eliminate background variance and spatiotemporal band‐pass filtering to suppress high‐frequency noise [57, 58].

Initial slopes (*k_raw_*) were obtained by performing spline interpolation on the original data and then taking the derivative. The raw fluorescence slopes (*k_raw_*) from each microwell were first calibrated to obtain the intrinsic catalytic rates (*k_corr_*). The statistical distribution of these *k* values across the population was then modeled (Supplementary Text). Core kinetic parameters—catalytic synthesis rate (*µ*, mean) and heterogeneity (*σ^2^*, variance)—were derived by fitting the rate distribution to a GMM using the scikit‐learn library in Python. Temporal synthesis variation (*θ*), defined as the temporal coefficient of variation for the mean rate (*µ_1_* to *µ_30_*), was calculated as in Equation (2).

#### 5.7.2 Biological Replicates and Reproducibility

All experiments were independently repeated at least three times (N ≥ 3) on different days with fresh reagents to ensure reproducibility.

#### 5.7.3 Data Screening

To ensure analytical integrity, rigorous spatial and temporal filters were applied during the early phase of signal acquisition. Theoretically, fluorescence intensities across the microwell array should exhibit baseline uniformity during initial reaction cycles. Therefore, microchambers exhibiting initial outlier intensities—defined as values deviating more than three times the standard deviation of initial baseline intensities from the global mean—were classified as non‐viable. Empty background wells and trajectories exhibiting poor linearity (R^2^< 0.90) within the regression window were excluded from downstream analysis, yielding typically >1000 highly confident single‐molecule kinetic traces per condition for final statistical modeling. This rigorous statistical filtering effectively eliminates the minor fraction of microwells containing two or more enzymes (which statistically represent ∼ 1.2% of the total wells at *λ* ≈ 0.15), ensuring that the analyzed kinetics strictly reflect single‐molecule events.

### 5.8 Quantification of Laser Intensity Effects

Based on the APA model (Supplementary Text 3), by utilizing a 10X objective (*NA_eff_* ≈ 0.02) to focus a 30 mW laser beam, a localized power density of approximately 20 kW/cm^2^ was achieved—exceeding the standard SMRT sequencing irradiance (approximately 1 kW/cm^2^) by 20‐fold. Under the linear reciprocity law of photon‐induced oxidative stress, 30‐min laser treatment at this intensity is kinetically equivalent to approximately 10 h of continuous sequential exposure, providing a high‐fidelity surrogate for multi‐generational motor failure analysis (Table S6).

The effects of laser irradiation were quantified by varying the irradiation duration. A laser system matching the spectral characteristics of the SMRT sequencer was employed. High‐purity enzyme samples (at the system final concentration) were aliquoted into transparent containers, including non‐irradiated controls. Experimental samples were exposed to a 30 mW laser beam (focused through the objective to a 10 µL) for 5, 10, or 30 min. To eliminate macroscopic thermal artifacts caused by high photon flux density, sample tubes were maintained in an ice‐water bath (∼4°C) throughout irradiation (Active Thermal Clamping). Immediately post‐irradiation, samples were diluted (4.34 × 10^6^‐fold) into reaction buffer to quench free radical propagation and dissipate residual thermal energy, ensuring that subsequent single‐molecule readouts predominantly reflected intrinsic enzymatic damage. Both treated and control samples were then evaluated using dSMAT. Data were systematically analyzed to compare activity shifts, relative rate changes, and laser dosage correlations. Laser safety protocols, including eye protection, were strictly followed.

### 5.9 Comparative Verification Calculation of Sequencing

We compared the dSMAT‐derived catalytic rate (*r_ca_* = 3.42 nt/s) with the data from the SMRT sequencing run. The Mode of the Local Base Rate in the sequencing data was 3.41 nt/s.

In addition, FWHM corresponding to heterogeneity(*σ^2^*) is calculated as:

(5) $$\mathrm{FWHM}=2\sigma \sqrt{2\;\mathrm{In}\;2}$$

## Author Contributions

**Jinze Li**: conceptualization, methodology, writing – review and editing, funding acquisition, supervision, validation. **Jia Yao**: software, formal analysis, data curation. **Xuefeng Wang**: investigation, validation, visualization, methodology. **Fuqiang Ma**: investigation, validation, methodology, project administration. **Wei Zhang**: writing – review and editing, methodology, conceptualization, project administration, funding acquisition, visualization, supervision, resources. **Anran Zheng**: conceptualization, methodology, writing – original draft, writing – review and editing, investigation, validation, formal analysis, visualization, funding acquisition. **Chuanyu Li**: conceptualization, funding acquisition, formal analysis, project administration. **Dongshu Li**: investigation, validation, data curation. **Qi Yang**: software, data curation, validation, formal analysis, visualization, writing – review and editing, writing – original draft, investigation. **Zhiqi Zhang**: supervision, resources, project administration. **Lianqun Zhou**: supervision, resources, project administration, visualization, funding acquisition, writing – review and editing, conceptualization, methodology. **Zhen Guo**: conceptualization, writing – review and editing, funding acquisition, formal analysis.

## Conflicts of Interest

The authors declare no conflicts of interest.

## Supporting information




**Supporting File 1**: advs76238‐sup‐0001‐SuppMat.docx.

**Supporting File 2**: advs76238‐sup‐0002‐DataFile.zip.

## Data Availability

The data that support the findings of this study are openly available in Google Drive at https://drive.google.com/drive/folders/1qDlbOekBtf8nWK5oNpDDOk8NitG31XAX?usp=sharing.
