# Supplementary material for: AI‐Assisted Digital Single‐Molecule Activity Tracker for Decoupling Intrinsic Heterogeneity from Photo‐Oxidative Damage in High‐Photon‐Flux Enzymology
Source: Adv Sci (Weinh). 2026 Jun 18:e76238. Online ahead of print. doi: 10.1002/advs.76238 (PMC13336840; doi:10.1002/advs.76238)
Supplement: Supplementary file 1 — Supporting File 1: advs76238‐sup‐0001‐SuppMat.docx. [file ADVS-9999-e76238-s002.docx]

**Supporting Information**

**AI-assisted digital single-molecule activity tracker for decoupling intrinsic heterogeneity from photo-oxidative damage in high-photon-flux enzymology**

Anran Zheng ^a,#^, Qi Yang ^a,#^, Jinze Li ^a,b^, Fuqiang Ma ^a,b^, Xuefeng Wang ^a^, Zhen Guo ^a,b^, Chuanyu Li ^a,b^, Dongshu Li ^a,b^ , Jia Yao ^a,b^ , Zhiqi Zhang ^a,b^, Wei Zhang ^a,b*^, Lianqun Zhou ^a,b*^

a CAS Key Lab of Bio-Medical Diagnostics, Suzhou Institute of Biomedical Engineering and Technology, Chinese Academy of Sciences, Suzhou, 215163, China

b School of Biomedical Engineering (Suzhou), Division of Life Sciences and Medicine, University of Science and Technology of China, Hefei 230026, China

Anran Zheng and Qi Yang contributed equally to this work.

Correspondence: Wei Zhang [(zhangw@sibet.ac.cn),](mailto:(zhangw@sibet.ac.cn),) Lianqun Zhou [(zhoulq@sibet.ac.cn)](mailto:(zhoulq@sibet.ac.cn)).

Supplementary Text

**1. Final Rate Computation**

The raw slope *k_raw_* (calculated from the linear regression of positive microwells within the sliding window) is calibrated to the absolute synthesis rate *r_ca_* (nt/s):

$\text{k}_{\text{corr}}\text{=}\frac{\text{k}_{\text{raw}}\text{-}\text{∇}_{\text{bg}}\left( \text{t} \right)}{\text{α}}$ Equation (S1)

Where:

*k_raw_*: The observed slope of fluorescence intensity (RFU/min).

*∇_bg_(t)*: The background drift rate (RFU/min) at time t, derived from the derivative of *F_bg_(t)*. Given the high stability of our system, this term is minimal but included for rigor.

*α*: The system response factor, measured as 2488.4 RFU/nM (equivalent to ~ 2.488 RFU/pM) based on bulk standard curve fitting (**Figure S1B**).

$\text{r}_{\text{ca}}\left( \text{μ} \right)\text{=}\text{μ}\text{×}\text{ϕ}_{\text{vol}}\text{×}\frac{\text{1}}{\text{60}}$ Equation (S2)

*Φ_vol_*: The geometric conversion factor bridging volumetric sub-picomolar increments back to absolute reaction events. In a 432 pL microwell, calculating absolute single-molecule product accrual yields an expected base constant coefficient of 260. Thus, equating from the scale equivalent to 1 pM product generated in situ calculates directly as:

($\text{ϕ}_{\text{vol}}\text{=1 }\text{p}\text{M}\text{×4.32×}\text{10}^{\text{-10}}\text{×6.022×}\text{10}^{\text{23}}\text{=260}$).

1/60: Unit conversion from minutes to seconds.

1. **Algorithm Model**

**2.1 Cross-Attention Mechanism**

To mitigate mechanical drift, we introduced a Cross-Attention block at the bottleneck stage that fuses visual features with spatial prompts. The Query (*Q*), Key (*K*), and Value (*V*) vectors are generated as follows:

The bottleneck feature map *F_img_* represents the dense visual information. To formulate the Query, *F_img_* is flattened along the spatial dimensions to form a sequence of visual tokens:

$\text{Q}\text{=}\text{Linear}\left( \text{Flatten}\left( \text{F}_{\text{img}} \right) \right)\text{∈}\text{R}^{\left( \text{h}\text{⋅}\text{w} \right)\text{×}\text{d}_{\text{model}}}$ Equation (S3)

Where dmodel is the embedding dimension.

The input point prompts (centroids of microwells from the previous frame) are represented as coordinate sets {(*x_1_ ,y_1_* ),...,(*x_N_ ,y_N_* )}. These coordinates first undergo Positional Encoding (using sinusoidal functions similar to Transformer architectures) to map low-dimensional coordinates to high-dimensional space. This is followed by a Multi-Layer Perceptron (MLP) to generate the prompt embeddings *E_prompt_*. The Keys and Values are derived from these prompt embeddings:

$\text{K}\text{=}\text{Linear}_{\text{K}}\left( \text{E}_{\text{prompt}} \right)\text{∈}\text{R}^{\text{N}\text{×}\text{d}_{\text{model}}}$ Equation (S4)

$\text{V}\text{=}\text{Linear}_{\text{V}}\left( \text{E}_{\text{prompt}} \right)\text{∈}\text{R}^{\text{N}\text{×}\text{d}_{\text{model}}}$ Equation (S5)

The Cross-Attention output focuses the network on the target microwell regions by calculating the similarity between image patches (*Q*) and prompt locations (*K*):

$\text{Attention}\left( \text{Q}\text{, }\text{K}\text{, }\text{V} \right)\text{=}\text{Softmax}\left( \frac{\text{Q}\text{K}^{\text{T}}}{\sqrt{\text{d}_{\text{model}}}} \right)\text{V}$ Equation (S6)

This output is then reshaped back to *h×w×C* and fed into the Decoder.

**2.2 Datasets Preparation**

We constructed a training dataset containing over 4000 microwell fluorescence images. The images were collected in five batches across two fluorescence channels: FAM and ROX. Each image contains 20,000 microwells, automatically labeled using ImageJ and manually selected and corrected. The training, validation, and test sets were in an 8:1:1 ratio. Online data augmentation was applied during training, including horizontal/vertical flipping and injecting Gaussian noise to simulate sensor noise.

1. **Determination of Equivalent Sequencing Time**

**3.1 Physics of Accelerated Photo-aging Model**

To characterize the photostability limit of polymerases, we defined a relative stress factor (A.F.) comparing experimental dSMAT irradiance (*I_exp_*) with a standard sequencing reference (*I_exp_* ≈ 1 kW/cm^2^).

System Numerical Aperture (*NA_eff_*), theoretical Spot Diameter (*d*), experimental irradiance (*I_exp_*) are calculated as:

$\text{NA}_{\text{eff}}\text{=}\frac{\text{r}_{\text{beam}}}{\text{f}}$ Equation (S7)

Where,

$\text{f}\text{=}\frac{\text{200}}{\text{Magnification}}$ Equation (S8)

For the 10X objective, *NA_eff_* is 0.015.

Derived from diffraction limits:

$\text{d}\text{≈}\frac{\text{λ}}{\text{2∙}\text{NA}_{\text{eff}}}$ Equation (S9)

For 532 nm, *d_10X_* ≈ 17.7 µm.

Calculating mean power density:

$\text{I}_{\text{exp}}\text{=}\frac{\text{P}}{\text{π}\text{∙}\left( \text{d}/\text{2} \right)^{\text{2}}}$ Equation (S10)

Given *P* = 30 mW, *I_exp_* for 10X mode reaches ~20 kW/cm^2^.

**3.2 Equivalent Time derivation**

The terminal equivalent time (*t_eq_*) on-sequencer is calculated as:

$\text{t}_{\text{eq}}\text{=}\text{t}_{\text{ill}}\text{×}\frac{\text{I}_{\text{exp}}}{\text{I}_{\text{ref}}}$ Equation (S11)

At 10 min illumination, *t_eq_* ≈ 3.33 h.

Since real-time SMRT trace analytics reveal an effective excitation duty cycle of 31.2% (**Data S6**), 3.33 h represents the accumulated high-energy dose of a standard 10-hour sequencing movie, establishing our 10-minute treatment as the comparable working equivalent.


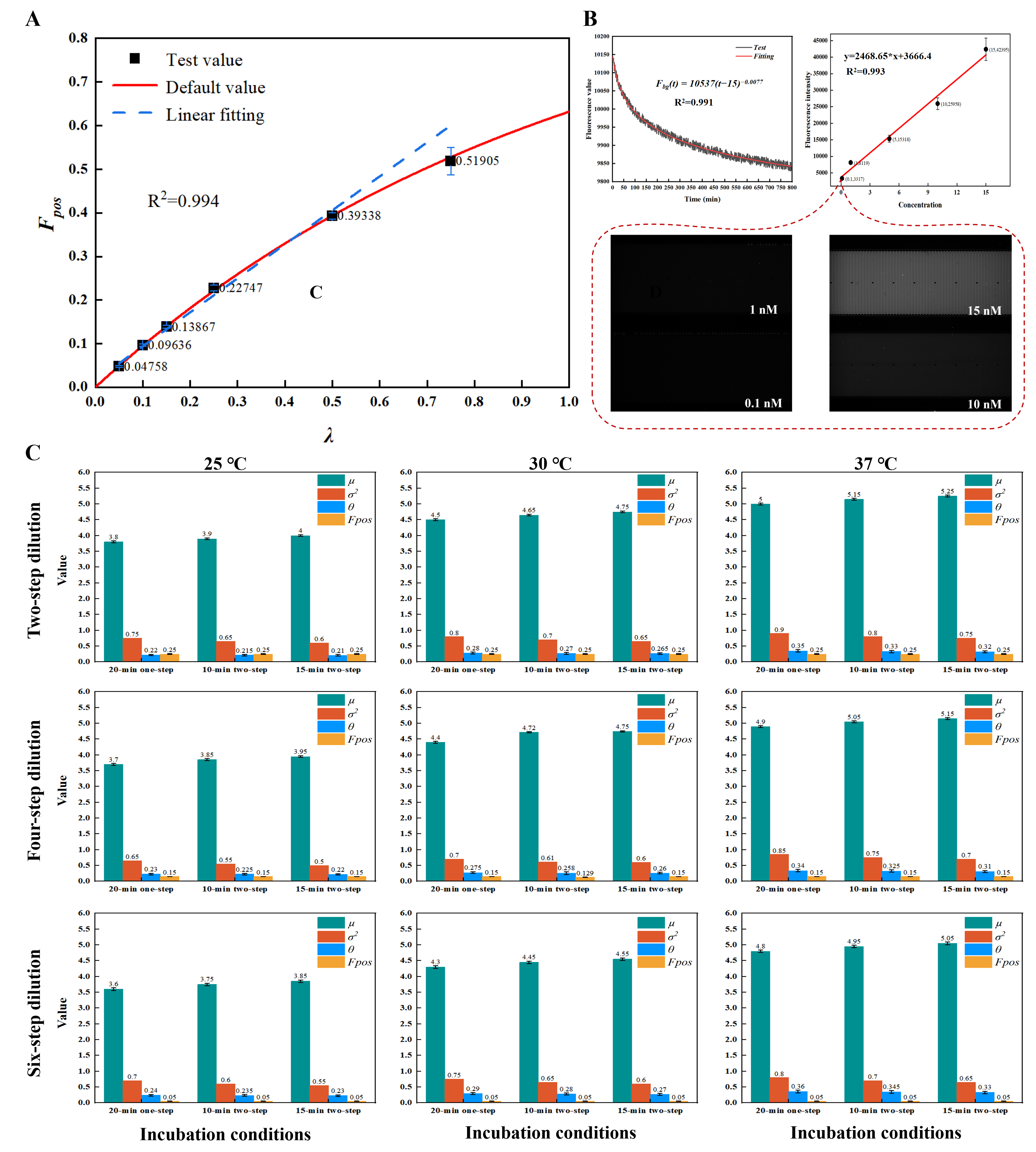


Figure S1. Reliability Analysis of dMAST Strategy. (A) Validation of single-molecule encapsulation efficiency. The experimentally measured fraction of positive micropores is plotted against the theoretical average occupancy (*λ*). Data points represent the mean ± s.d. from three independent experiments. The solid red line represents the theoretical curve for an ideal Poisson distribution (*F_pos_ = 1−e^−λ^*). The blue dashed line represents the linear correlation between the theoretical prediction (or expected values) and the experimental (measured) outcomes. The excellent agreement confirms stochastic single-molecule partitioning (R^2^ = 0.994). (B) Calibration of background dynamics and system response factor. Left: Temporal dynamics of the background fluorescence baseline. The black trace represents the mean fluorescence intensity of negative control microwells (without DNA polymerase, N > 1000 wells) monitored over 900 minutes at 30 ℃. The red solid line indicates the best-fit power-law decay function: *F_bg_(t) = 10537(t−15)^−0.0077^* (R^2^ = 0.991). The instantaneous derivative of this function (*∇_bg_*) determines the background drift rate used in Equation (1). Right: System response standard curve relating fluorescence output to molecular product concentration. The plot shows the fluorescence intensity of Molecular Beacon-Target complexes at varying concentrations (0, 0.1, 1, 5, 10, and 15 nM) measured under conditions identical to the dSMAT assay. Data points represent the mean ±s.d. of three independent replicates. The blue solid line represents the linear regression fit (R^2^ = 0.986). The derived slope, α = 2488.4 a.u.nM^−1^, serves as the system response factor to calibrate the raw kinetic slope (*k_raw_*) into absolute nucleotide synthesis rates. (C) The optimizations of critical operational steps and procedural settings.


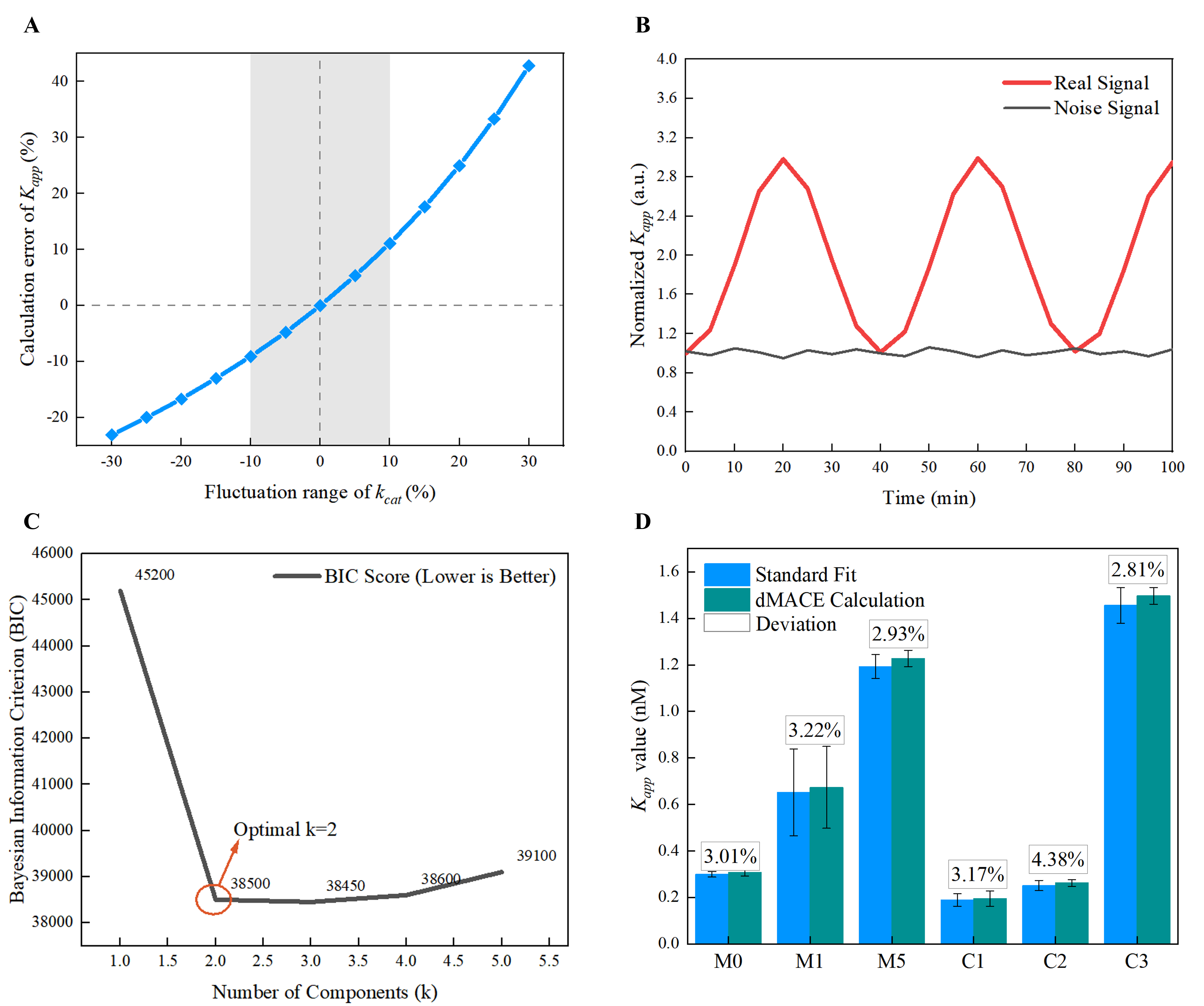


Figure S2. Sensitivity analysis simulation of the *K_app_* calculation model. (A) The quantitative relationship between fluctuations in intrinsic catalytic rate (*k_cat_*) and the resulting theoretical deviation in the calculated apparent kinetic efficiency index (*K_app_*). The gray shaded area indicates that typical stochastic fluctuations in *k_ca_*_t_ (±10%) result in limited deviation in *K_app_*. (B) A comparative simulation over a representative 100-min observation window. The black line (Artifact) shows the minor *K_app_* fluctuations (~ 10%) that would be caused solely by random *k_cat_* noise. In contrast, the red line (Observed Signal) models the large-amplitude dynamics (> 2-fold change) typical of experimentally observed affinity shifts. (C) The result of BIC Analysis. (D) Validation of the single-point affinity estimation strategy via population-level consistency check. The bar chart illustrates the quantitative comparison between the standard Michaelis constant (*K_m_*, fit) and the Equation-derived *K_app_*. Left: The standard *K_m_* value derived from the hyperbolic fitting of the M0 polymerase titration curve. Right: The population-averaged *K_app_* calculated using the single-point estimation formula at a substrate concentration of 0.25 nM. The minimal deviation (indicated in gray) confirms the mathematical validity of the *K_app_* metric for approximating substrate affinity under the experimental conditions.


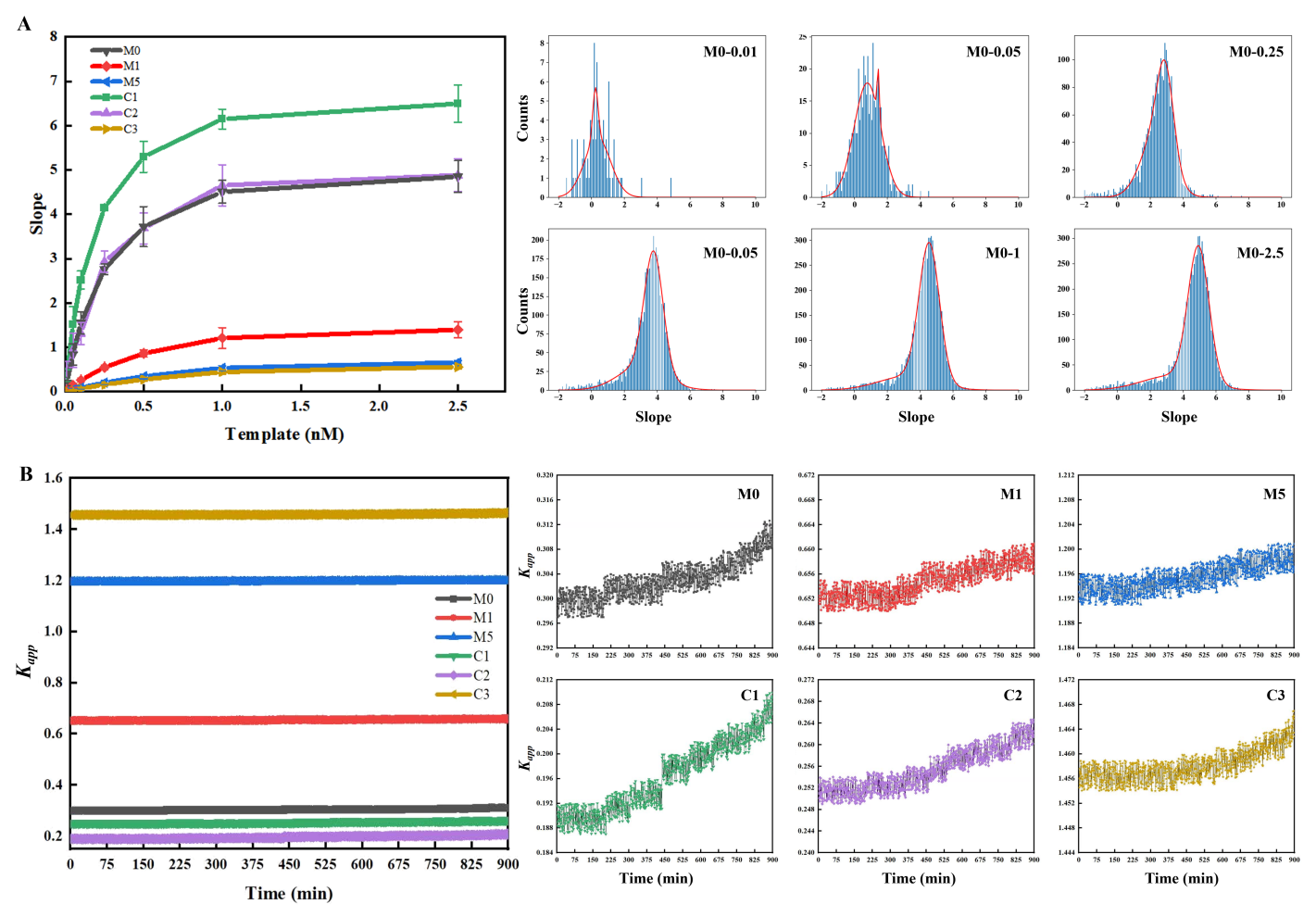


Figure S3. Apparent kinetic efficiency index (*K_app_*) changes and situations of six phi29 DNA polymerase (M0/M1/M5/C1/C2/C3) assessed by dSMAT method. (A) The left panel displays the catalytic synthesis capacity of six distinct DNA polymerases at varying concentrations measured at the 30-minute time point, while the right panel shows the slope distribution of the wild-type enzyme M0 across these concentrations; (B) The left panel illustrates the temporal evolution of *K_app_* values over a 900-minute period, with the right panel providing magnified views of individual enzyme trajectories.


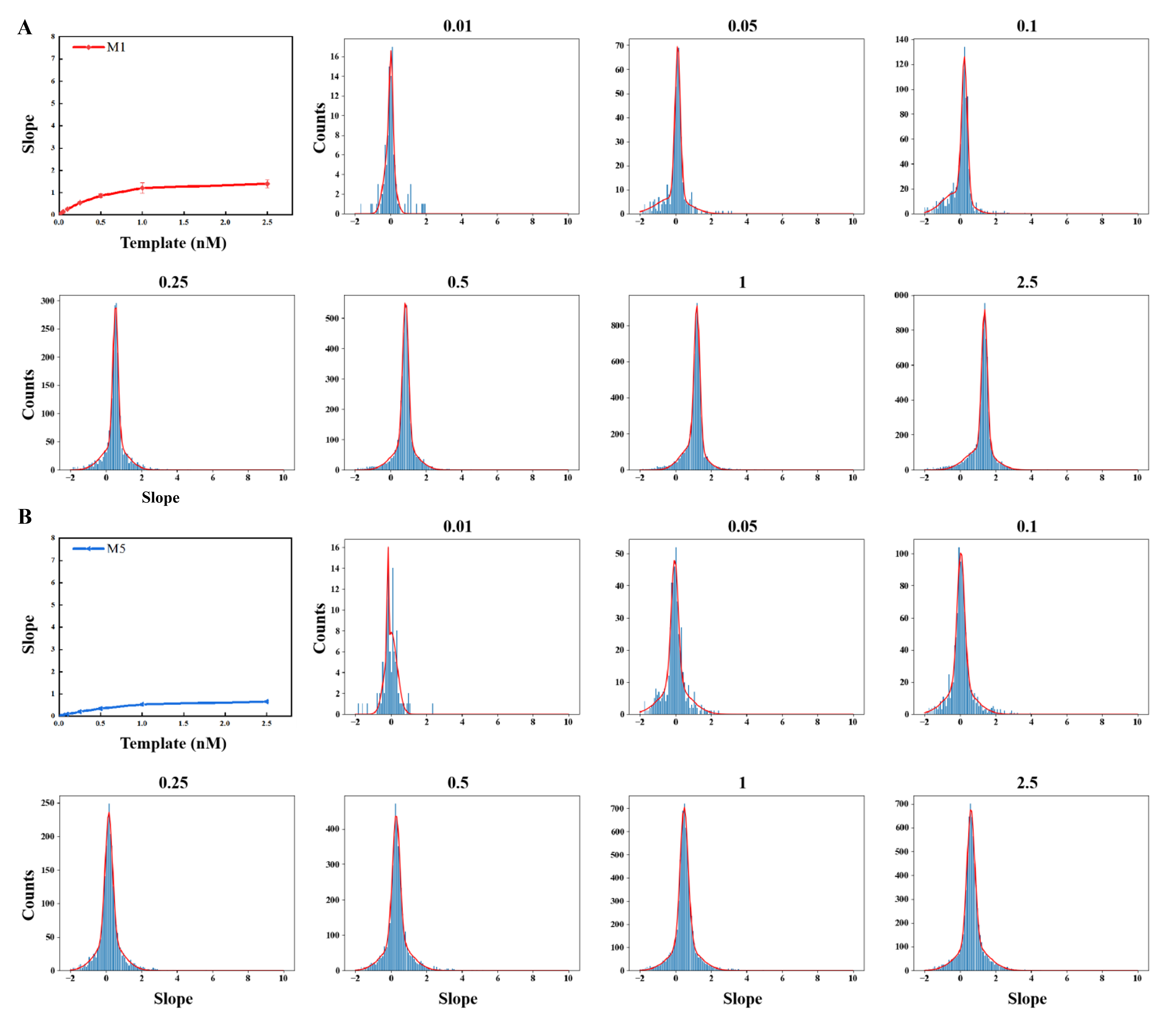


**Figure S4. Instantaneous Apparent Kinetic Efficiency (*K_app_*) Distributions for Mutant Enzymes.** (A) M1 enzyme. (B) M5 enzyme.


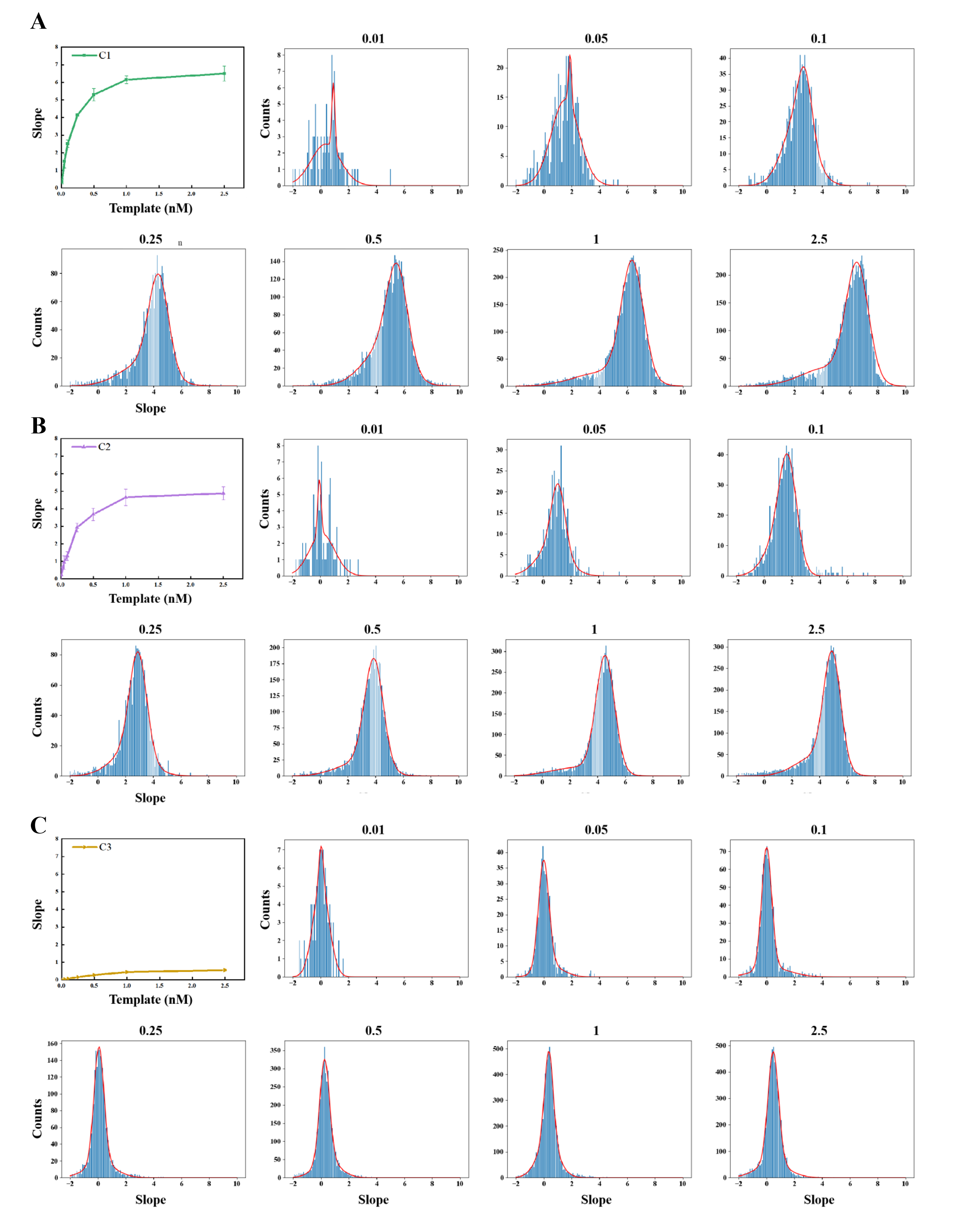


**Figure S5. Instantaneous Apparent Kinetic Efficiency (*K_app_*) Distributions for Commercial Enzyme.** (A) C1 enzyme. (B) C2 enzyme. (C) C3 enzyme.


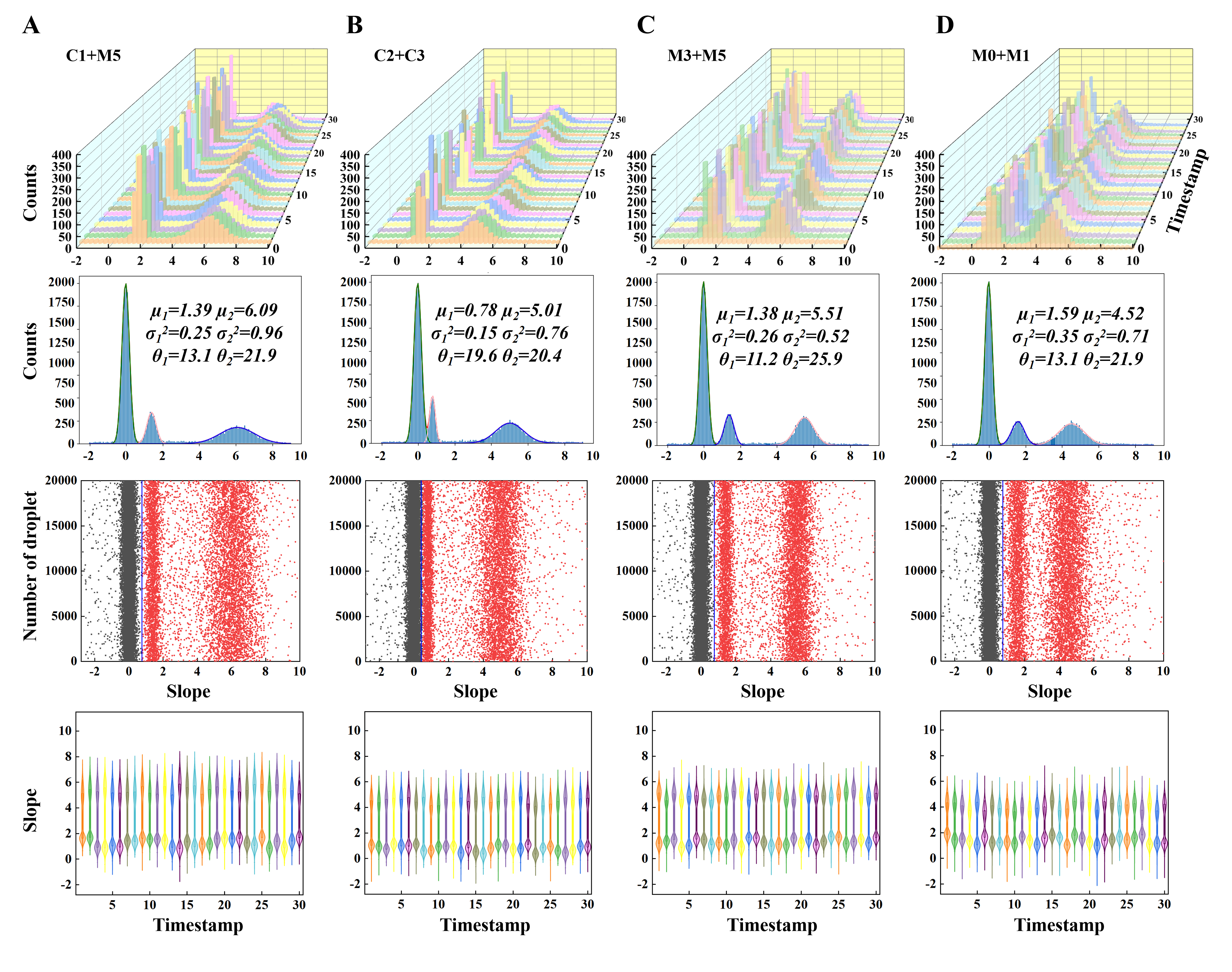


**Figure S6. Resolving subpopulations in mixed enzyme samples using dSMAT.** The dSMAT platform was employed to deconvolve the multi-activity profiles of heterogeneous mixtures containing two distinct phi29 DNA polymerase variants per sample. The analyzed mixtures are: (A) Commercial enzyme C1 mixed with mutant M5. Two populations are resolved: a high-activity, heterogeneous population (*μ*₂ = 6.09, *σ*₂^2^ = 0.96, *θ*₂ = 21.9%) and a low-activity, homogeneous population (*μ*₁ = 1.39, *σ*₁^2^ = 0.25, *θ*₁ = 13.1%), consistent with the individual profiles of C1 and M5, respectively. (B) Commercial enzyme C2 mixed with C3. Two populations are resolved: a high-activity population (*μ*₂ = 5.01, *σ*₂^2^ = 0.76, *θ*₂ = 20.4%) and a low-activity population (*μ*₁ = 0.78, *σ*₁^2^ = 0.15, *θ*₁ = 19.6%). (C) Mutant M3 mixed with M5. (D)Wild-type M0 mixed with mutant M1. The dSMAT strategy effectively identifies and quantifies individual enzyme species within a mixture based solely on multi-activity fingerprints, without the need for physical separation. This highlights its potential for screening complex enzyme libraries and assessing the heterogeneity of commercial enzyme preparations.


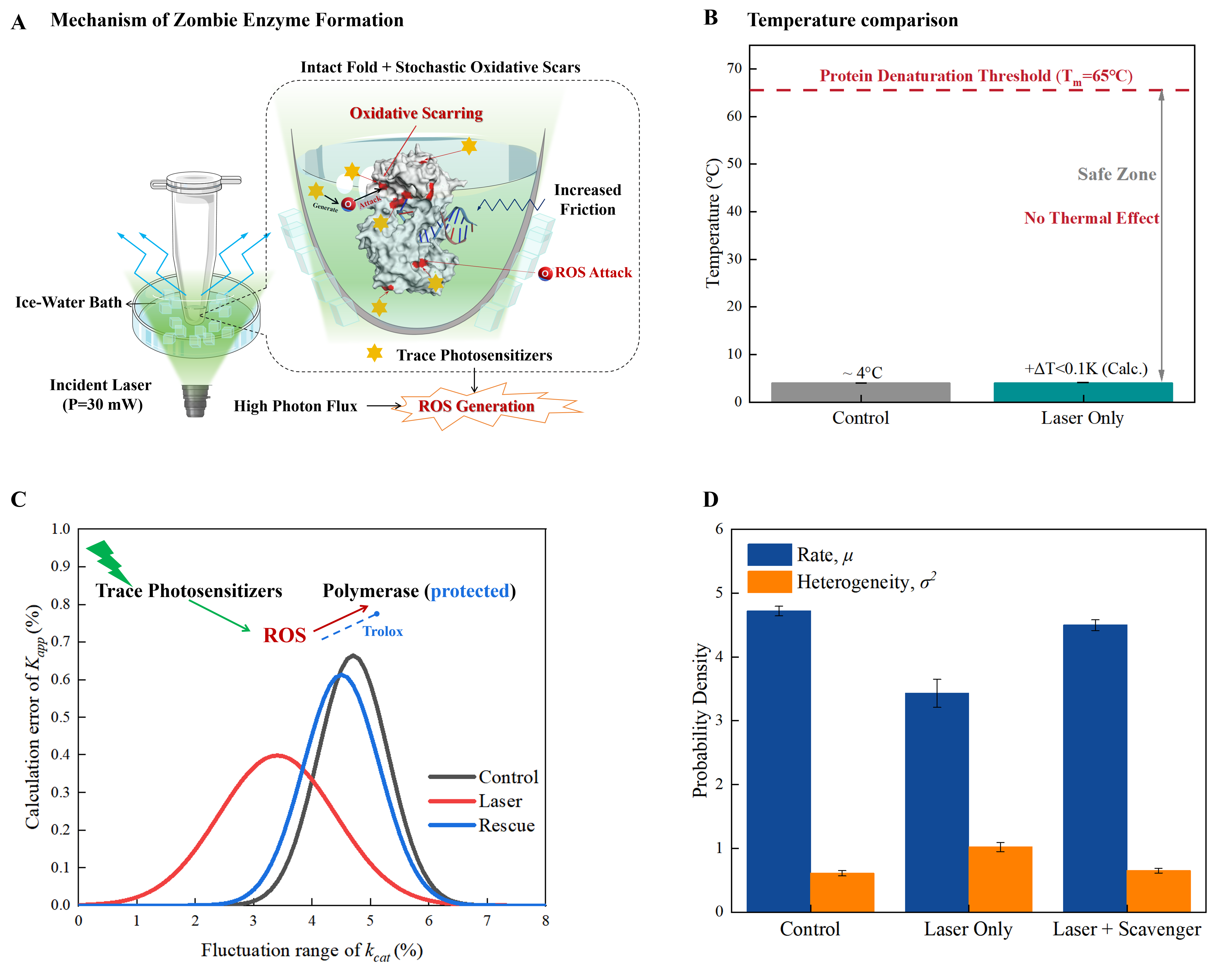


**Figure S7. Validation of non-thermal mechanism via active thermal clamping and specific ROS scavenging.** (A) Schematic of the setup: The enzyme stock (10 μL) was irradiated (30 mW) while immersed in an ice-water bath (4 °C). (B) Comparative visualization: The Y-axis represents absolute temperature. Even with the theoretical laser-induced heating (< 0.1 °C), the sample temperature remains near 4 °C (blue bar), which is ~ 60 °C below the enzyme's denaturation threshold (*T_m_* ≈ 65℃, red dashed line). This large temperature margin provides robust evidence that the observed kinetic changes are driven by photochemical oxidation (ROS attack) and not by thermal denaturation. (C) The distribution profiles of single-molecule catalytic rates (*μ*) for phi29 DNA polymerase (M0) under three different conditions: Control (untreated, gray curve), Laser Only (exposed to 30 mW laser for 30 min, red curve), and Laser add Scavenger (exposed to 30 mW laser in the presence of 1 mM Trolox, green curve). The Laser Only group exhibits a distinct leftward shift (decreased activity) and peak broadening (increased heterogeneity). In contrast, the addition of the Trolox effectively restores the rate distribution profile to a state resembling the control, demonstrating a rescue effect. Proposed mechanism: DTT acts as a sacrificial electron donor in the presence of trace metals, generating singlet oxygen and superoxide radicals. (D) Quantitative comparison of the catalytic synthesis rate (*μ*) and kinetic heterogeneity (*σ*^2^) across the three groups. The data show that the Trolox significantly inhibits the laser-induced degradation of *μ* and the increase in *σ*^2^. Since antioxidants specifically neutralize oxidative radicals without affecting thermal dissipation.


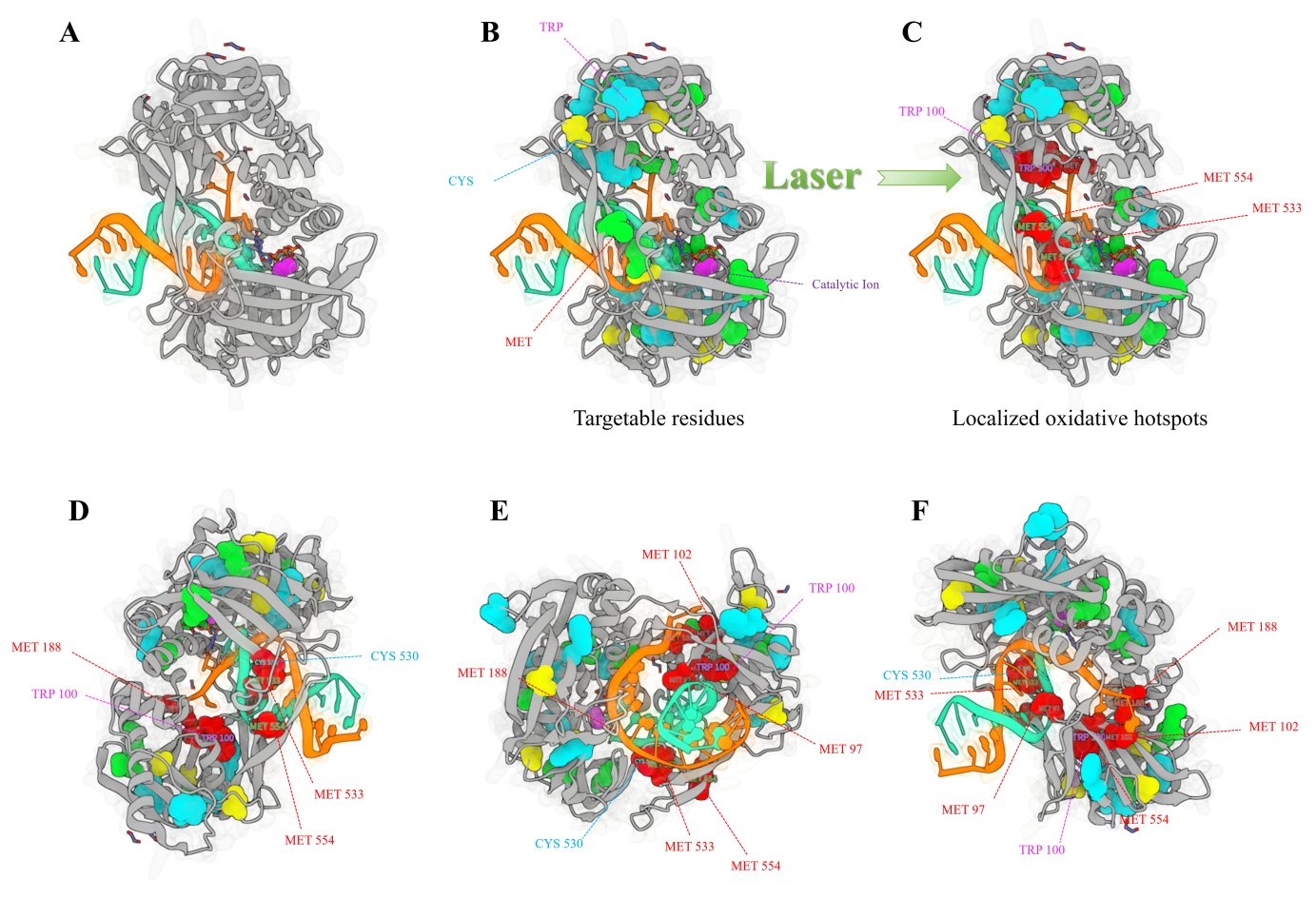


**Figure S8. Mechanistic rationalization of oxidative scarring through structural hotspots mapping.** (A) Ternary complex of phi29 DNA polymerase (gray, include catalytic ion magenta sphere) with template DNA (orange, PDB: 2PYL). (B) Global distribution of total oxidation-prone residues (Met, green; Trp, yellow; Cys, cyan). (C) Schematic of laser-induced scarring: the arrow denotes a 30-min laser dose under infinite heat-sink clamping. Specific labels (red residues) highlight the residues located at the critical DNA translocation cleft. (D–F) Multiple orientations cross-sectional views demonstrating that a significant population of sensitive residues is strategically localized within the DNA binding cleft. The stochastic transition of these hydrophobic residues into bulky polar adducts (e.g., Met-sulfoxide) during irradiation creates high-friction zones, expanding the inter-molecular heterogeneity (*σ^2^*) and reducing the average velocity (*μ*) measured in the dSMAT assay.


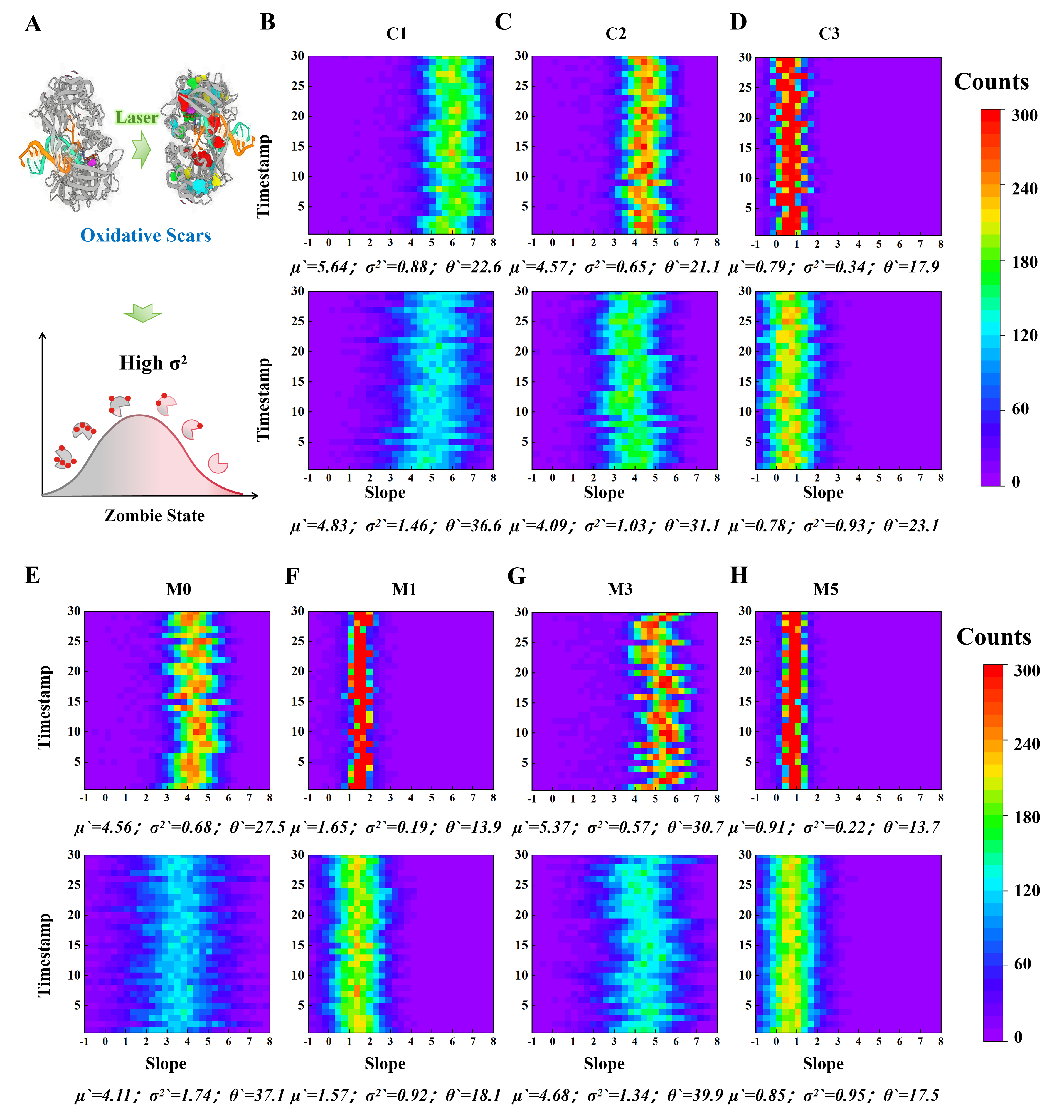


**Figure S9. Kinetic profiling of different phi29 DNA polymerases after laser treatment (5/20 min) based on the dSMAT.** (A) Mechanistic model of oxidative scarring. Fundamentally different from thermal denaturation, which involves a binary all-or-none transition, high-intensity laser irradiation induces the cumulative accumulation of oxidative modifications on solvent-accessible surface residues. As exposure time increases (from 5 to 20 min), this stochastic damage creates steric friction, transforming the native homogeneous population into a broad, kinetically impaired heterogeneous kinetic state (high *σ*^2^, conceptually modeled as an oxidatively scarred, sub-active ensemble (referred to as a "zombie-like" state)). (B–D) Time-resolved single-molecule kinetic profiling of commercial polymerase variants (C1, C2, and C3). (E–H) Time-resolved kinetic profiling of wild-type and engineered mutants (M0, M1, M3, and M5). Heatmaps display the distribution of catalytic rates (Slope) across the population over time (Y-axis: Timestamp within the assay).


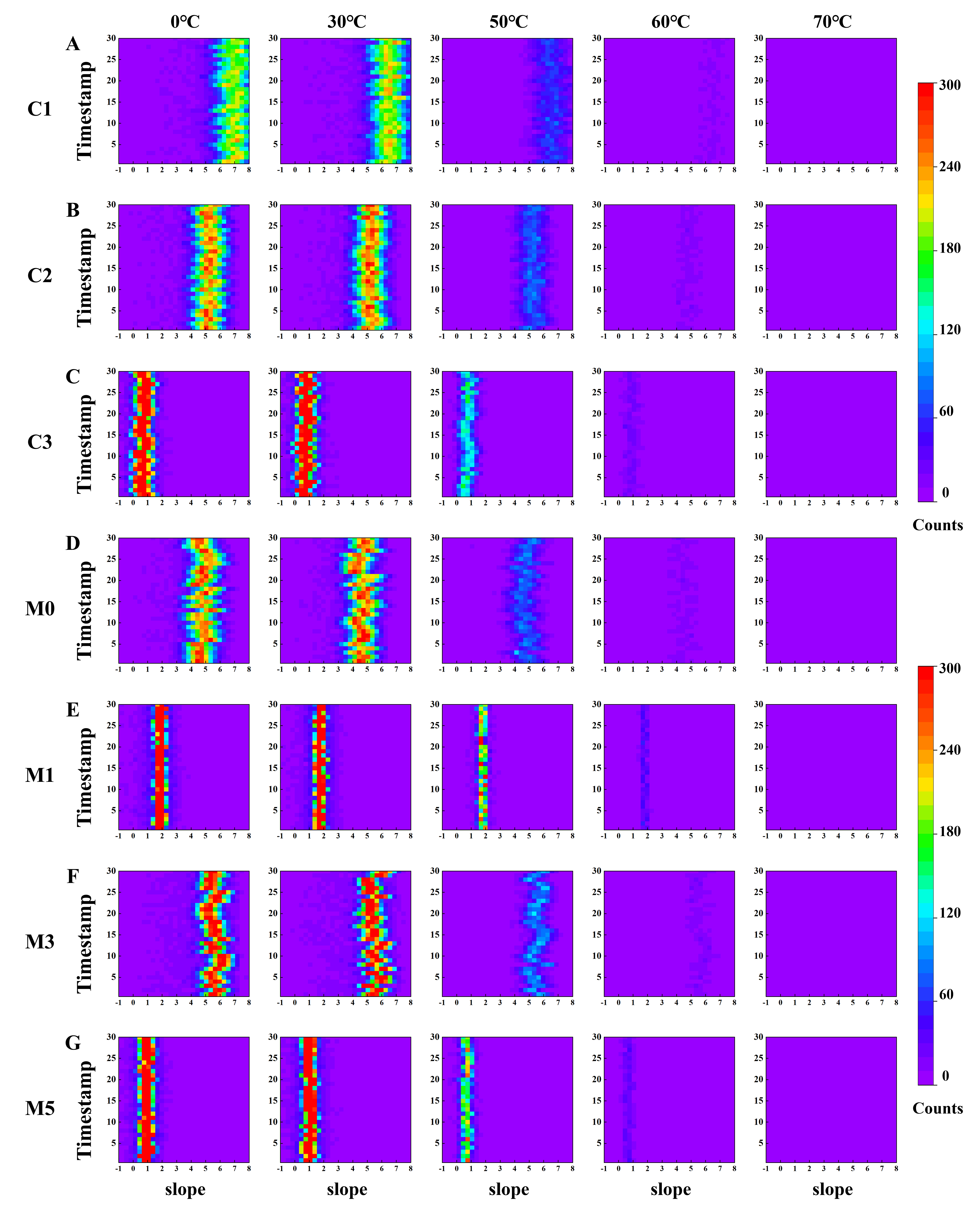


**Figure S10. Kinetic profiling of different phi29 DNA polymerases after heat treatment (0/30/50/60/70 ℃) based on the dSMAT.** (A–C) Time-resolved single-molecule kinetic profiling of commercial polymerase variants (C1, C2, and C3). (D–G) Time-resolved kinetic profiling of wild-type and engineered mutants (M0, M1, M3, and M5). Heatmaps display the distribution of catalytic rates (Slope) across the population over time (Y-axis: Timestamp within the assay).


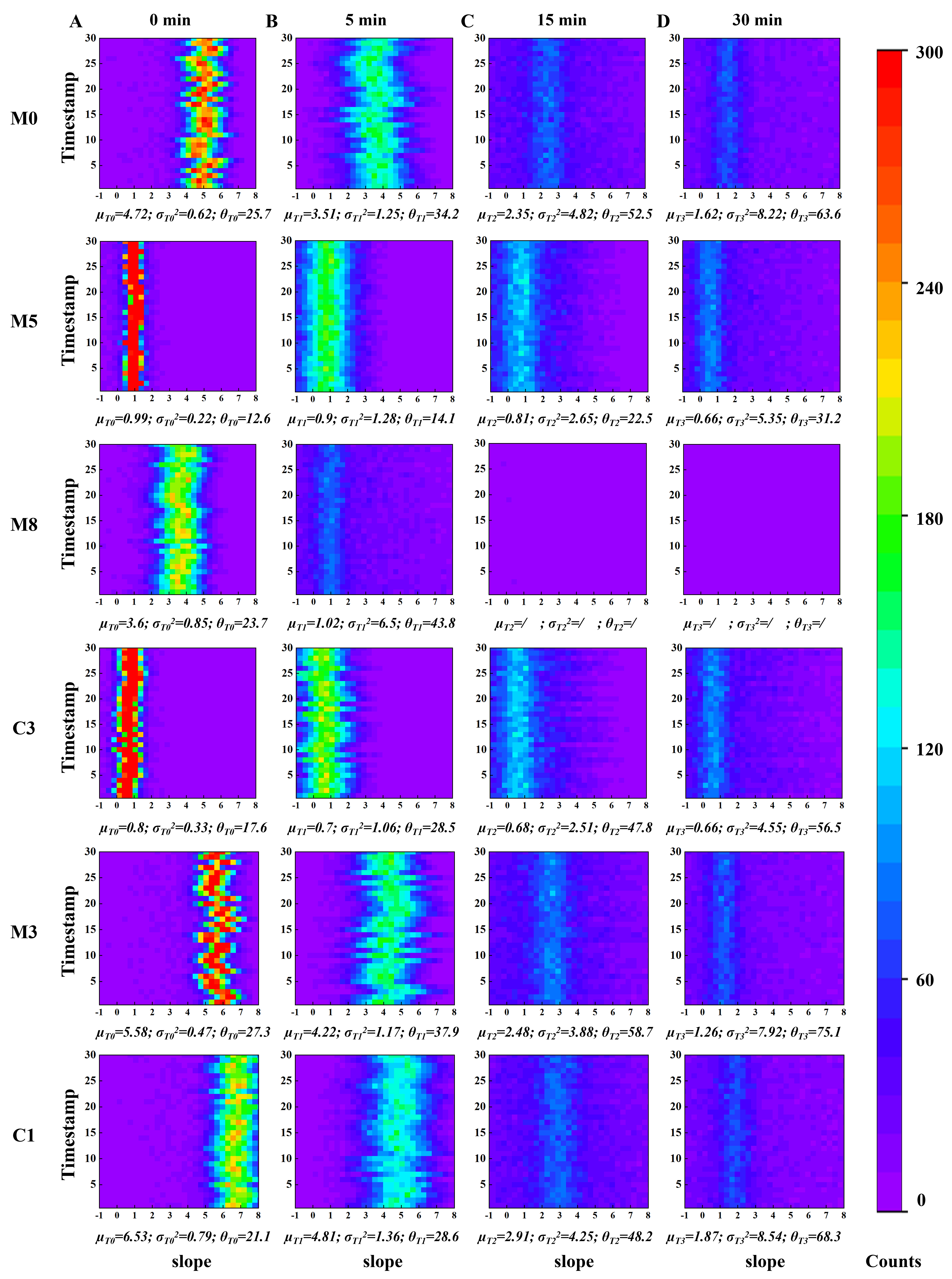


**Figure S11. Kinetic profiling of diverse phi29 DNA polymerases under elevated laser stress (40 mW).** (A–D) Spatiotemporal heatmaps illustrating the dynamic distribution of catalytic rates for M0 (wild-type), M5 (engineered resistant mutant), M8 (obsolete non-resistant mutant), C3 (commercial variant (resistant)), M3 (other mutant) and C1 (commercial variant (non-resistant)). The rows represent varying laser exposure durations (0, 5, 15, and 30 min). The X-axis denotes the catalytic rate (slope), the Y-axis represents the assay progression (timestamp).


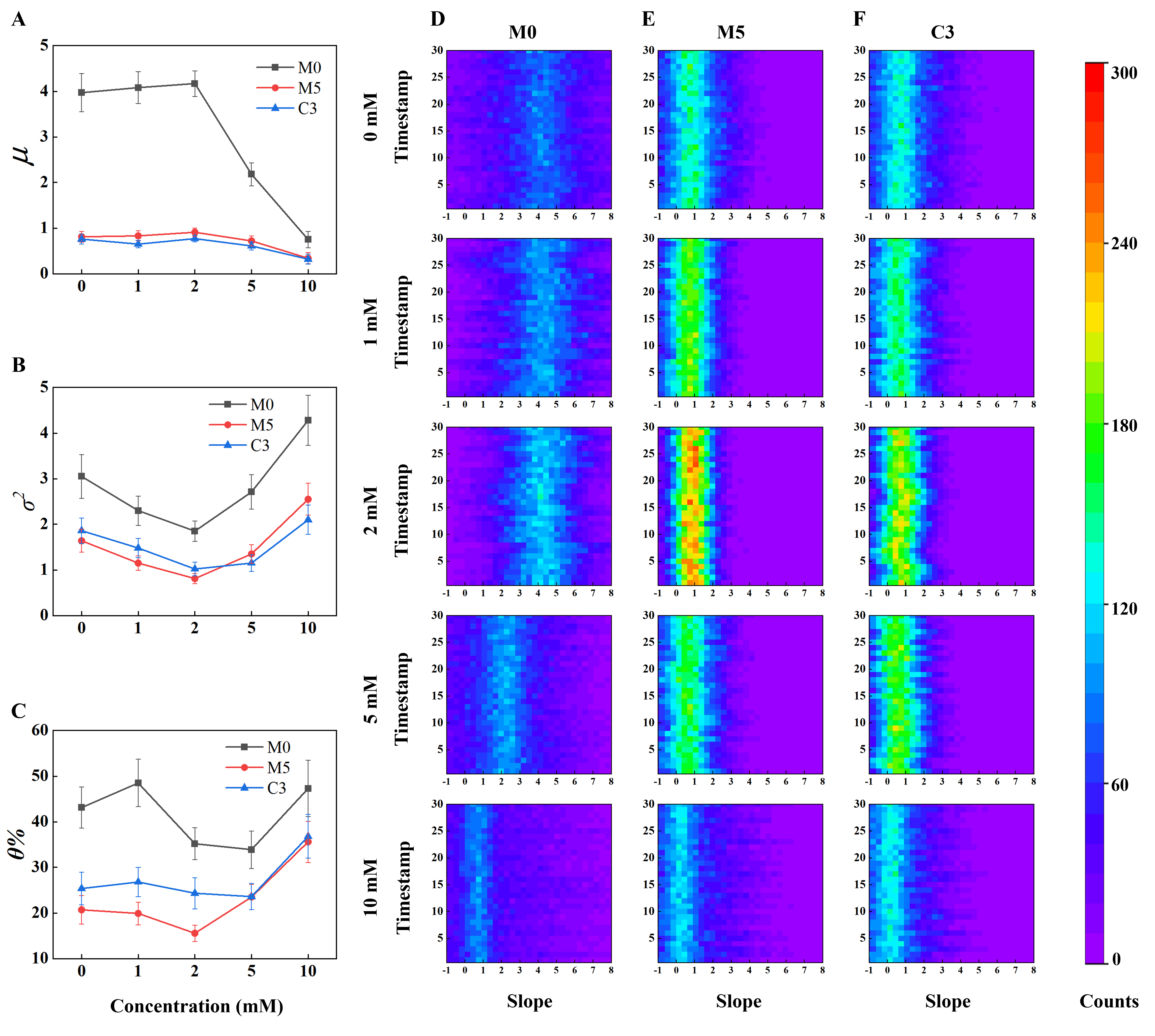


**Figure S12. Preliminary evaluation of Trolox concentration gradients on single-molecule polymerase kinetics.** The kinetic parameters of wild-type (M0), engineered mutant (M5), and commercial (C3) phi29 DNA polymerases were evaluated under a fixed 1.0 mM DTT and 30-min continuous laser stress. (A–C) Quantitative trajectories of the overarching catalytic rate (*μ*), intrinsic heterogeneity (*σ^2^*), and temporal synthesis fluctuation (*θ* %) across varying Trolox concentrations (0, 1, 2, 5, and 10 mM). (D–F) Time-resolved single-molecule kinetic heatmaps illustrating the dynamic distribution of catalytic rates for M0, M5, and C3.


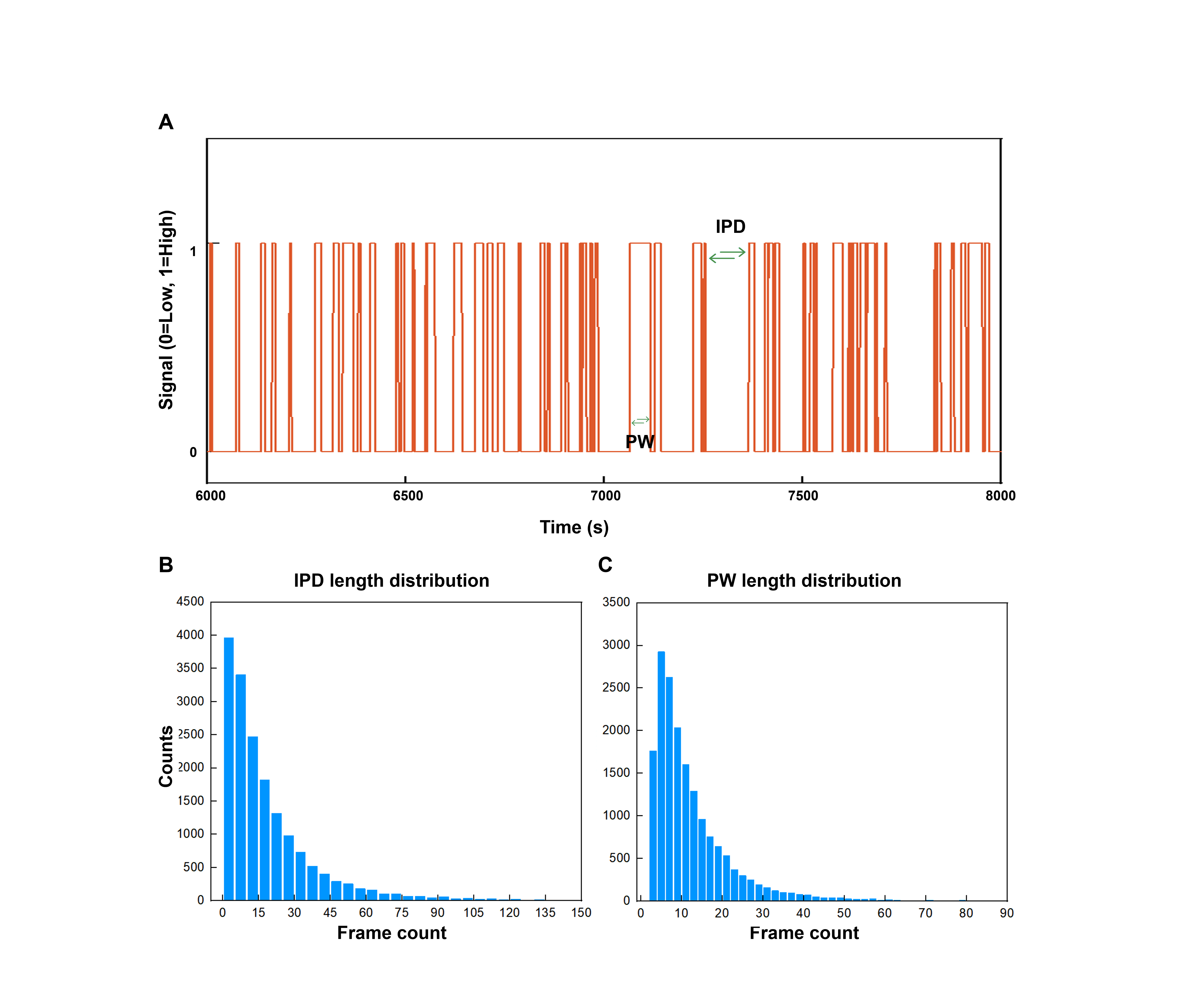


**Figure S13. Definition and statistical distribution of single-molecule kinetic parameters derived from SMRT sequencing traces.** (A) Representative binary trace of a single-molecule sequencing read. The signal states correspond to nucleotide incorporation events (High/1) and inter-incorporation pause (Low/0). Pulse Width (PW) denotes the duration of the fluorescence emission during synthesis, while Inter-Pulse Duration (IPD) represents the kinetic wait time between successive events. The IPD is inversely proportional to the catalytic rate (*μ*) characterized in dSMAT. (B) Frequency distribution histogram of IPD lengths (measured in camera frames) for the commercial C3 polymerase. The distribution reflects the stochastic nature of enzyme turnover. (C) Frequency distribution histogram of PW lengths (measured in frames).

Table S1. Theoretical calculation based on Poisson distribution

| Theoretical *λ* | Theoretical *F_pos_* | Test | Effective microwell | Signal points | Actual *F_pos_* | Actual *λ* |
| --- | --- | --- | --- | --- | --- | --- |
| 0.05 | 0.0488 | 1 | 20458 | 990 | 0.0484 | 0.0496 |
|  |  | 2 | 20469 | 1015 | 0.0496 | 0.0509 |
|  |  | 3 | 20471 | 916 | 0.0447 | 0.0458 |
| Mean ± SD | | 0.04758 ± 0.00252 | | | | |
| 0.1 | 0.0952 | 1 | 20465 | 1917 | 0.0937 | 0.0984 |
|  |  | 2 | 20472 | 2006 | 0.0980 | 0.1031 |
|  |  | 3 | 20476 | 1995 | 0.0974 | 0.1025 |
| Mean ± SD | | 0.09636 ± 0.00235 | | | | |
| 0.15 | 0.1393 | 1 | 20456 | 2860 | 0.1398 | 0.1506 |
|  |  | 2 | 20459 | 2769 | 0.1353 | 0.1454 |
|  |  | 3 | 20463 | 2882 | 0.1408 | 0.1518 |
| Mean ± SD | | 0.13867 ± 0.00292 | | | | |
| 0.25 | 0.2212 | 1 | 20468 | 4515 | 0.2206 | 0.2492 |
|  |  | 2 | 20464 | 4652 | 0.2273 | 0.2579 |
|  |  | 3 | 20470 | 4800 | 0.2345 | 0.2672 |
| Mean ± SD | | 0.22747 ± 0.00695 | | | | |
| 0.5 | 0.3935 | 1 | 20471 | 8183 | 0.3997 | 0.5104 |
|  |  | 2 | 20468 | 8087 | 0.3951 | 0.5027 |
|  |  | 3 | 20473 | 7888 | 0.3853 | 0.4866 |
| Mean ± SD | | 0.39338 ± 0.00738 | | | | |
| 0.75 | 0.5276 | 1 | 20459 | 11089 | 0.5420 | 0.7809 |
|  |  | 2 | 20466 | 9890 | 0.4832 | 0.6602 |
|  |  | 3 | 20476 | 10891 | 0.5319 | 0.7591 |
| Mean ± SD | | 0.51905 ± 0.03142 | | | | |

Table S2. The sequences of the molecular beacon and the template

| Type | Sequence (5’-3’) |
| --- | --- |
| Template | GTA TCT CTG GAC GGG AGT CCT GGT TTT AAT CCT GTG AGA TTC TGA GAA GAG CAT AGT TAC GGT AGC TGC CAT CGT CTG TTG TTC TCC GAC GAA CAT AGT ATA AGG CAA TAT GAG AAG GTC AAT |
| Primer | GCA CGA GGT CCA GAG ATA CAT TGA CCT TCT CCC CAC CAG |
| Molecular beacon | CAC GCC GGT AGC TGC CAT CGT CTG TTG TTT GCG TG (5’-FAM; 3’-BHQ1) |

Table S~~3~~. Theoretical calculation based on Poisson distribution

| Calculation Step | Value & Calculation |
| --- | --- |
| 1. Initial complex concentration | *C_i_* = 2.5 nM = 2.5×10^−9^ mol/L |
| 2. Single microwell sample loading volume | *V* = 432 pL = 4.32×10^−10^ L |
| 3. Target *λ* parameter | *λ* = 0.15 |
| 4. Required target concentration | *C_t_* = *λ / V*×*N_A_* = 0.15/2.6015×10^14^ |
|  | *C_t_* ≈ 5.764×10^−16^ mol/L |
| 5. Total dilution factor (*DF*) | *DF* = *C_i_/C_t_* ≈ 4.337×10^6^ |
| 6. Dilution scheme | Four-step serial dilution: |
|  | 1. 1:100 (into buffer) |
|  | 2. 1:100 (from step 1) |
|  | 3. 1:20 (from step 2) |
|  | 4. 1:20 (from step 3) |
| 7. Diluted concentration | *C_d_* = *C_i_/DF = 6.25*×10^−16^ mol/L |
| 8. Diluted *λ* parameter | *λ* = *C_d_* ×*V* × *N_A_* = 0.1626 |
| 9. Expected well occupancy | *P*(0) = *e*^−0.1626^≈0.850 |
|  | *P*(1) = 0.1626 × *e*^−0.1626^ ≈ 0.138 |
|  | *P*(≥ 2) ≈ 0.012 |
| 10. Expected positive fraction | *F_pos_* = 1−*P*(0) ≈ 0.15 |

Table S4. Preparation of the reaction system and process flow

| dSMAT | RCA system | Initial concentration | Final concentration | volume |
| --- | --- | --- | --- | --- |
| 1 | Template | 1 ng/µL | 0.1 ng/µL | 1 µL |
|  | Primers | 10 µM | 1 µM | 2.5 µL |
|  | ddH_2_O | - | | 6.5 µL |
|  | Total | | | 10 µL^*^ |
| 2 | product | - |  | 7 µL |
|  | phi29Buffer | 10X | 1X | 2 µL |
|  | dNTP | 5 mM | 0.5 mM | 2 µL |
|  | MB | 2 µM | 0.2 µM | 2 µL |
|  | BSA | 20 mg/mL | 2 mg/mL | 2 µL |
|  | F127 | 0.1% | 0.01% | 2 µL |
|  | phi29 | 10 U/µL | 0.5 U/µL | 1 µL |
|  | ddH_2_O | - | | 2 µL |
|  | Total | | | 20 µL^**^ |

^*^Note: 1 μL was diluted 4.337×10^6^ fold (four-step) to obtain the product

^**^ Note: Sample injected 9 μL and sealed 15 μL 30 °C 1 min shoot once for 900 cycles, FAM channel

Table S5. Classification Accuracy and Resolution of Mixed Enzyme Subpopulations

| Mixture Type | Component | Ground Truth（*µ* from Pure Enzyme） | GMM Resolved(*µ* from Mixture) | Recovery Accuracy |
| --- | --- | --- | --- | --- |
| C1 + M5 | Component 1 (M5) | 0.99 | 1.39 | 71.20% |
|  | Component 2 (C1) | 5.33 | 6.09 | 87.50% |
| C2 + C3 | Component 1 (C3) | 0.8 | 0.78 | 97.50% |
|  | Component 2 (C2) | 5.07 | 5.01 | 98.80% |
| M3 + M5 | Component 1 (M5) | 0.99 | 1.38 | 71.70% |
|  | Component 2 (M3) | 5.58 | 5.51 | 98.70% |

Table S6. Characterization of the Accelerated Photo-aging and Sequential Workload Equivalency

| **Objective Mode** | ***NA****eff* | **Spot**  **Diameter (*d*,μm)** | **Irradiance (*I****exp*, kW/cm2) | **Acceleration**  **Factor (*AF*)** | ***t****ill*= 10 min  **Equiv. (*t****eq*) | ***t****ill*= 30 min  **Equiv. (*t****eq*) |
| --- | --- | --- | --- | --- | --- | --- |
| 4 × | 0.006 | ~ 40 | ~ 2.5 | 2.5 fold | 25 min | 75 min |
| 10 × | 0.015 | ~ 17.7 | ~ 20.0 | 20.0 fold | 200 min (3.33 h) | 600 min (10 h) |
| 20 × | 0.03 | ~ 8 | ~ 80 | 80 fold | 13.3 h | 40 h |
| 60 × | 0.09 | ~ 2.7 | ~ 720 | 720 fold | 120 h | 360 h |

Data S1. (separate file)

Data visualized in Figure 3.

Data S2. (separate file)

Data visualized in Figure 4.

Data S3. (separate file)

Data visualized in Figure 5.

Data S4. (separate file)

Data visualized in Figure 6 and Figure S9.

Data S5. (separate file)

Data visualized in Figure 7.

Data S6. (separate file)

Data visualized in Figure 8.

Data S7. (separate file)

Data visualized in Figure S10.

Data S8. (separate file)

Data visualized in Figure S11.

Data S9. (separate file)

Data visualized in Figure S12.

Data S10. (separate file)

Statistical result of different phi29 DNA polymerases under different treatment.
